# Spinal cord injury dysregulates fibro-adipogenic progenitors miRNAs signaling to promote neurogenic heterotopic ossifications

**DOI:** 10.1038/s42003-023-05316-w

**Published:** 2023-09-12

**Authors:** Jules Gueguen, Dorothée Girard, Bastien Rival, Juliette Fernandez, Marie-Emmanuelle Goriot, Sébastien Banzet

**Affiliations:** 1 Institut de Recherche Biomédicale des Armées, 92140 Clamart, France; 2 INSERM UMR-MD-1197, 92140 Clamart, France

**Keywords:** Stem-cell research, Mechanisms of disease, Muscle stem cells, Trauma

## Abstract

Neurogenic heterotopic ossifications are intramuscular bone formations developing following central nervous system injury. The pathophysiology is poorly understood and current treatments for this debilitating condition remain unsatisfying. Here we explored the role of miRNAs in a clinically relevant mouse model that combines muscle and spinal cord injury, and in patients’ cells. We found an osteo-suppressive miRNAs response in injured muscle that was hindered when the spinal cord injury was associated. In isolated fibro-adipogenic progenitors from damaged muscle (cells at the origin of ossification), spinal cord injury induced a downregulation of osteo-suppressive miRNAs while osteogenic markers were overexpressed. The overexpression of selected miRNAs in patient’s fibro-adipogenic progenitors inhibited mineralization and osteo-chondrogenic markers in vitro. Altogether, we highlighted an osteo-suppressive mechanism involving multiple miRNAs in response to muscle injury that prevents osteogenic commitment which is ablated by the neurologic lesion in heterotopic ossification pathogenesis. This provides new research hypotheses for preventive treatments.

## Abstract (web summary)

miRNome analysis of skeletal muscle in a mouse model of neurogenic heterotopic ossification and human FAPs in vitro reveal that a fine-tuned balance between osteogenic and osteo-suppressive miRNAs drive FAPs osteogenic differentiation.

## Introduction

Neurogenic Heterotopic Ossifications (NHOs) are ectopic extra-skeletal bone formations, that occur after central nervous system injury, primarily spinal cord injury (SCI), traumatic brain injury and stroke^1–3^. NHOs develop at the expense of skeletal muscle tissue, are located around major joints and can impair limb mobility^4,5^. NHOs can induce joint ankylosis, nerve and vascular compressions and severe pain. Surgical resection is currently the most efficient and reliable treatment^5,6^. Up to 30% of SCI and 20% of traumatic brain injury patients develop NHOs, leading to comorbidities and compromising patient’s rehabilitation^6,7^.

NHOs development involves a process of endochondral ossification and can lead to a mature bone containing a functional hematopoietic niche^8–10^. Fibro-adipogenic progenitors (FAPs) are muscle interstitium resident mesenchymal stromal cells that are major cellular actors in HOs development and specifically in NHOs^11–13^. Physiologically, FAPs support myogenic progenitors (MPs) proliferation and myoblastic differentiation upon muscle repair but can also lead to fibrosis under pathological conditions^14–17^. FAPs directly participate in bone fracture repair, exhibiting previously unsuspected physiological osteogenic capabilities^18^. Muscle regeneration after acute injury is orchestrated by MPs activation, proliferation, differentiation into myoblasts and fusion to form new myofibers^19–21^. Lineage tracing experiments have shown that MPs do not contribute to HOs nor NHOs but muscle regeneration seems to be compromised^13,22^. Inflammation is a key mechanism in NHOs pathogenesis. Pro-inflammatory cytokines and macrophages, but not neutrophils, are involved and mandatory to drive FAPs towards an osteogenic fate^23,24^. Central nervous system injury triggers local and systemic inflammatory response^25,26^. Pro-inflammatory cytokines Interleukin-1 and Oncostatin M directly support NHOs pathogenesis in a mouse model and in vitro with patient’s muscle progenitors^8,27^. Despite all previous studies, some aspects of NHOs pathogenesis are still unclear, specifically the impact of the central nervous system lesion on FAPs and MPs signalling and fate.

MiRNAs are small non-coding single stranded RNA that post-transcriptionally regulate gene expression by binding and silencing their mRNA target^28,29^. Numerous miRNAs are physiologically involved in bone development and repair processes, they regulate osteoblast proliferation/differentiation or hypertrophic maturation of chondrocytes by targeting key molecular actors such as *Runx2*, *Sox9*, *Osx*, *Bmp2*, *Atf4* or their repressors^30–32^. While cellular actors and numerous cytokines have been thoroughly investigated in HOs, the potential involvement of miRNAs is still poorly described. MiR-17-5p and miR-203 regulate HOs in ankylosing spondylarthritis and in a non-neurogenic HO model, respectively^33,34^. In a small cohort of human traumatized muscle samples, miRNA dysregulation was found in patients developing HOs and 2 myomiRs were proposed to contribute to ectopic ossification^35^. Although these studies demonstrate the involvement of miRNAs in HOs formation, their possible role in NHOs remains unclear.

We hypothesized that miRNAs could participate to NHOs formation and studied their expression in our NHOs mouse model combining SCI and acute cardiotoxin (CDTX) muscle injury^23^. In this model, SCI or muscle injury alone do not elicit NHOs, both injuries are needed. To understand why injured muscle fails to regenerate and engages in osteo-chondrogenic processes following SCI, we performed a large miRNAs profiling at early stages, in skeletal muscle tissue. Candidate miRNAs were then studied in mice FAPs and MPs isolated from our model. Dysregulated miRNAs were further studied in vitro in osteogenic differentiation conditions in FAPs isolated from NHOs patients muscle sample and functional assays were performed on human FAPs with the most relevant ones.

In this study, we identified early osteo-suppressive miRNAs downregulation in FAPs concomitant to myogenic miRNAs downregulation in MPs in the NHOs mouse model. We also confirmed that overexpression of these osteo-suppressive miRNAs could attenuate mineralization and osteogenic markers expression from human FAPs.

## Results

### SCI induces mineralization and exacerbates osteo-chondrogenic markers expression in CDTX damaged muscle

**Fig. 1:**
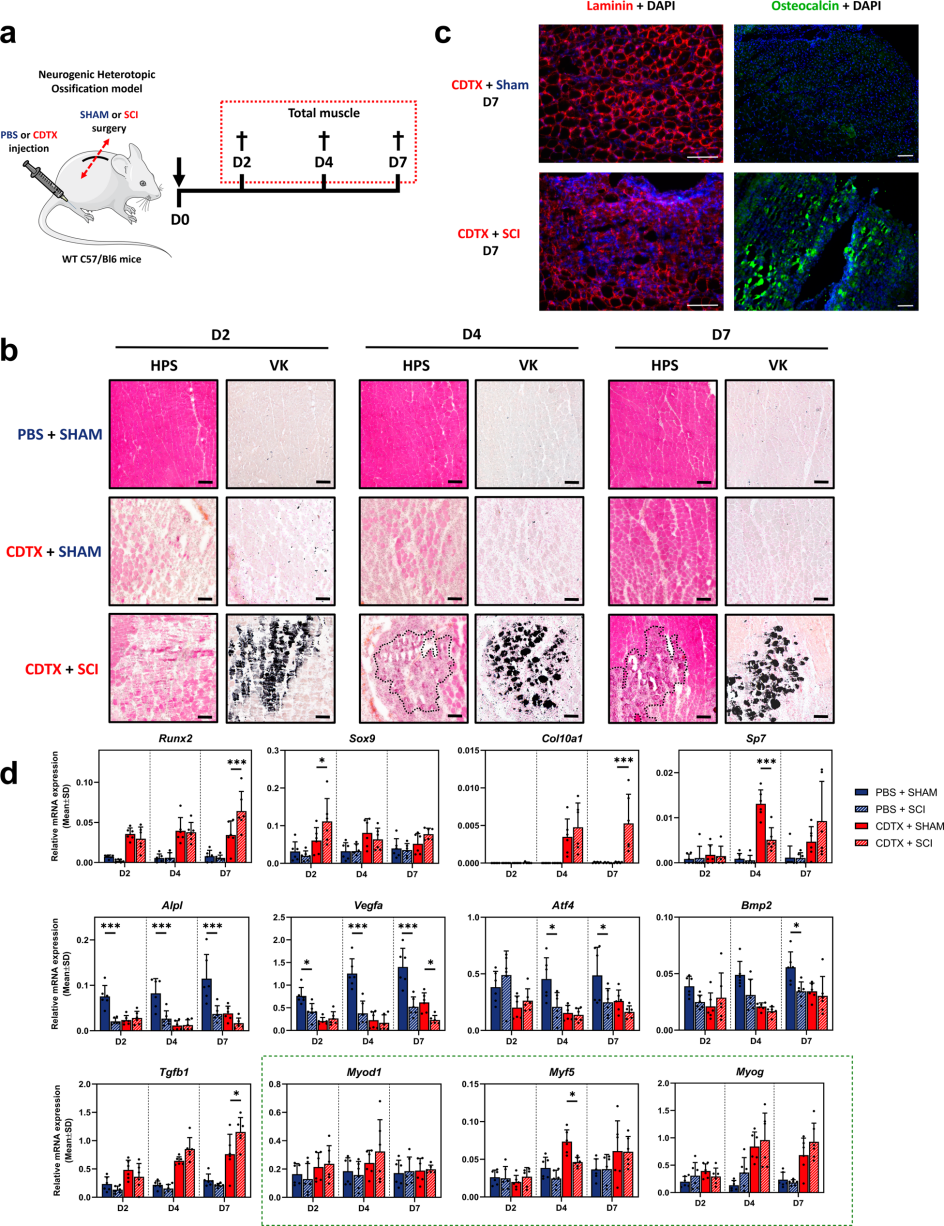
SCI induces mineralization and exacerbates osteo-chondrogenic markers expression in CDTX damaged muscle. **a** NHOs mouse model combines muscle injury (CDTX) and central nervous system damage (SCI) to induce intramuscular ectopic bone formation. PBS and SHAM surgery are controls. Muscles were collected at 2, 4 and 7 days after induction from four experimental groups: PBS + SHAM, PBS + SCI, CDTX + SHAM, CDTX + SCI. This figure panel were partly generated using Servier Medical Art, provided by Servier, licensed under a Creative Commons Attribution 3.0 unported license. **b** Representative transversal gastrocnemius cryosections of PBS + SHAM (top panel), CDTX + SHAM (middle panel) and CDTX + SCI (bottom panel) stained with Hematoxylin Phloxine Saffron (HPS) or Von Kossa (VK) counterstained with nuclear fast red at day 2, 4 and 7 (*n* = 6 in all groups). Mineralization nodules (black dotted line) appear in dark purple with HPS staining or in black with VK staining (magnification ×10, scale bar = 100 µm) (*n* = 6 in each group). **c** Representative laminin (left panel, magnification ×20, scale bar = 100 µm) and osteocalcin (right panel, magnification ×10, scale bar = 100 µm) immunostaining with DAPI co-staining on CDTX + SHAM or CDTX + SCI gastrocnemius transversal cryosections 7 days after injury (*n* = 6 in each group). **d** Relative mRNAs expression levels measured by RT-qPCR from whole muscle in PBS + SHAM, PBS + SCI, CDTX + SHAM, CDTX + SCI at days 2, 4 and 7. Myogenic markers are framed in green. Values are normalized on three reference genes and histograms represent mean ± standard deviation (SD). Each dot represents independent biological samples from different mouse muscles (*n* = 6). Statistical differences between PBS + SHAM/PBS + SCI and CDTX + SHAM/CDTX + SCI were calculated using two-way ANOVA with Tukey post hoc correction for multiple comparisons (*P < 0.05, **P < 0.01, ***P < 0.001).

To investigate the role of miRNAs in NHOs, we used our mouse model that combines muscle injury by intramuscular injection of cardiotoxin (CDTX) and concomitant spinal cord injury (SCI) to generate ectopic ossification, or their controls (intramuscular PBS injection and SHAM surgery)^23^ (Fig. 1a). We collected gastrocnemius muscle 2, 4 or 7 days later. In injured muscle, the hematoxylin phloxine saffron staining showed classical myofiber necrosis with massive mononuclear cells infiltration at D2, D4 and regenerative myofibers at D7 (Fig. 1b, top and middle panel). No mineralization was observed with Von Kossa staining with CDTX alone (Fig. 1b) nor with SCI alone (Supplementary Fig. 1). Undamaged muscle from SCI mice displayed myofibers atrophy at D7 (Supplementary Fig. 1). SCI combined with CDTX injection induced myofibers necrosis and cell infiltration at D2, D4 and D7 (Fig. 1b). Von Kossa staining showed calcified necrotic fibers (D2, D4) and nodules (D7), consistent with previous findings^23^. Immunostaining analysis showed disrupted laminin staining and impaired muscle regeneration, concomitant to extracellular osteocalcin and collagen III deposition in SCI + CDTX muscles, demonstrating that osteogenic differentiation occurred (Fig. 1c and Supplementary Fig. 1). These results confirm that both muscle and spinal cord injuries are mandatory to the development of NHOs.

The kinetics of signaling pathways activation involved in the early stages of NHOs development is still poorly described, therefore we investigated the expression of osteo-chondrogenic and myogenic markers at the mRNA level in muscle tissue at D2, D4, D7 in PBS + SHAM, PBS + SCI, CDTX + SHAM, CDTX + SCI muscles (Fig. 1d). We observed increased expression of osteo-chondrogenic markers *Runx2*, *Sox9* and *Sp7* induced by CDTX injection while CDTX + SCI exacerbated *Sox9* expression compared to CDTX alone at D2. Moreover, *Runx2*, *Col10a1* and *Tgfb1* expressions were increased at D7 in CDTX + SCI muscle compared to CDTX alone. Surprisingly, *Alpl*, *Vegfa, Atf4* and *Bmp2* expression levels were lowered in both CDTX conditions and PBS + SCI at each time point compared to PBS + SHAM. *Myf5* myogenic marker expression was significantly lowered in CDTX + SCI muscle compared to CDTX alone at D4 while *Myod1* and *Myog* expression did not change significantly (Fig. 1d).

### Muscle injury triggers osteogenic miRNAs expression while SCI impairs and delays osteo-suppressive miRNAs response after muscle damage

**Fig. 2:**
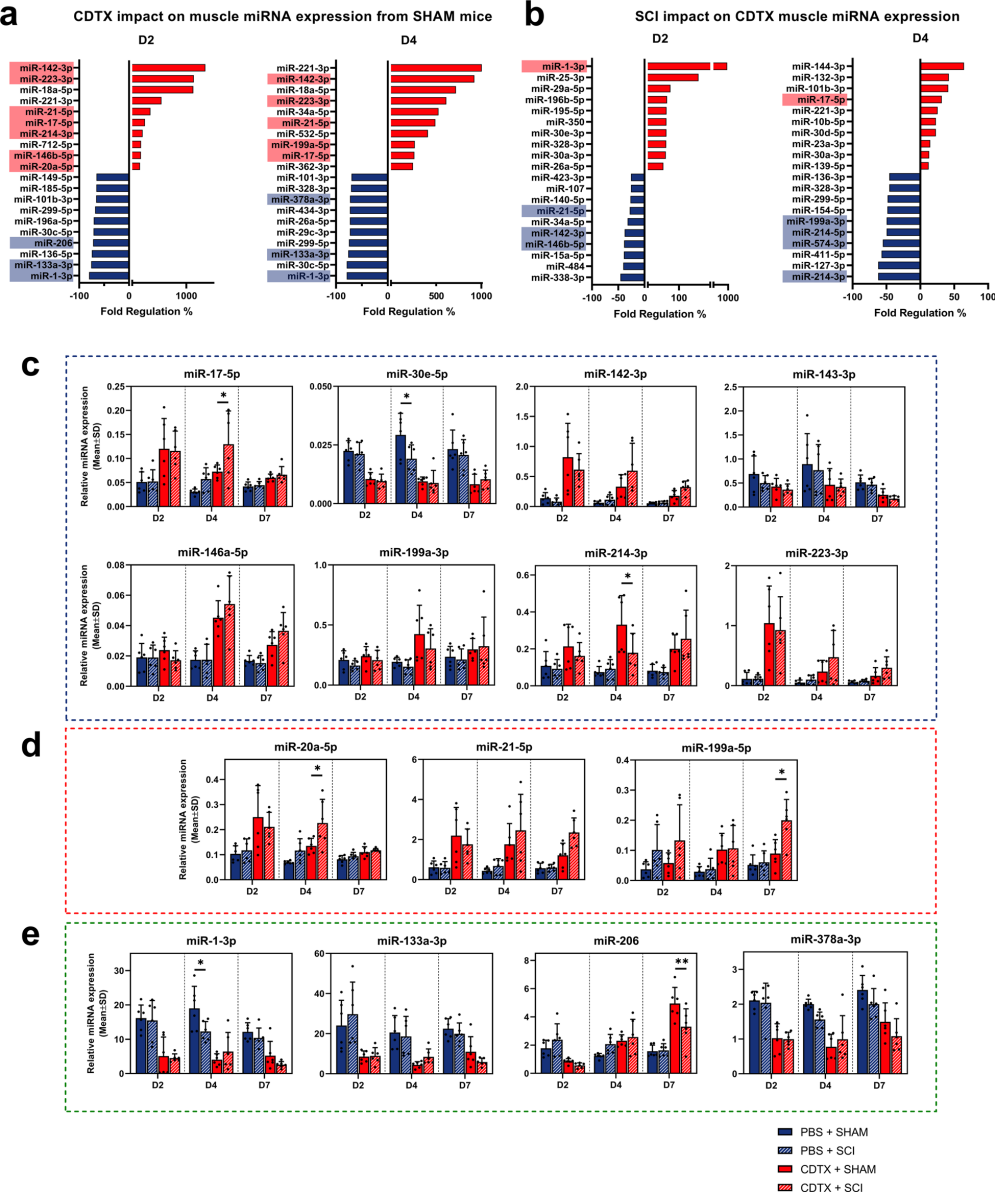
Muscle injury triggers osteogenic miRNAs expression while SCI impairs and delays osteo-suppressive miRNAs response after muscle damage. RT-qPCR analysis on whole muscle at day 2 and 4 presenting top ten most overexpressed and underexpressed miRNAs from **a** CDTX + SHAM compared to PBS + SHAM, **b** CDTX + SCI compared to CDTX + SHAM. MiRNAs involved in osteogenic, inflammatory, and myogenic processes are highlighted in red (upregulated) or blue (downregulated). A threshold was established to exclude miRNAs expressed later than 32 Cp. Relative miRNAs expression levels measured by RT-qPCR from whole muscle PBS + SHAM, PBS + SCI, CDTX + SHAM, CDTX + SCI at days 2, 4 and 7 for **c** osteo-suppressive miRNAs, **d** osteogenic miRNAs and **e** myomiRs. Histograms represent mean ± standard deviation (SD). Each dot represents independent biological samples from different mouse muscles (*n* = 6). Statistical differences between PBS + SHAM/ PBS + SCI and CDTX + SHAM/CDTX + SCI were calculated using two-way ANOVA with Tukey post hoc correction for multiple comparisons (*P < 0.05, **P < 0.01, ***P < 0.001).

Next, we investigated global miRNA expression in injured muscle at the early time points D2 and D4 using a RT-qPCR profiling analysis (Fig. 2) performed on pooled samples. CDTX injury alone induced the overexpression of several osteogenic miRNAs: miR-21-5p, miR-20a-5p and miR-199a-5p and osteo-suppressive miRNAs: miR-142-3p, miR-223-3p, miR-17-5p and miR-214-3p at D2 and D4 after muscle injury (Fig. 2a). CDTX injury also decreased the expression of multiples muscle-specific miRNAs (myomiR) such as miR-1-3p, miR-133a-3p, miR-206 and miR-378a-3p (Fig. 2a). Interestingly, the upregulation of several osteo-suppressive miRNAs was ablated by SCI (Fig. 2b).

We next further explored the expression of relevant miRNAs candidates selected based on our miRNAs profiling (Fig. 2a, b and Supplementary Fig. 2) crossed with extensive literature analysis on osteo-chondrogenic, myogenic and inflammation related miRNAs. These candidates were measured on individual samples by RT-qPCR. In CDTX-injured muscle, we observed an upregulation of osteo-suppressive miR-17-5p, miR-142-3p, miR-146a-5p, miR-199a-3p, miR-214-3p and miR-223-3p compared to PBS injected muscle (Fig. 2c). Among these osteo-suppressive miRNAs, miR-17-5p expression was further upregulated at D4 in CDTX + SCI compared to CDTX alone whereas miR-214-3p expression was significantly downregulated. Osteo-suppressive miR-30e-5p expression was significantly reduced by both SCI and CDTX injury at D4, while miR-143-3p expression was downregulated in CDTX-injured muscle (Fig. 2c). Moreover, osteogenic miRNAs miR-20a-5p and miR-21-5p were upregulated in CDTX muscle with miR-20a-5p and miR-199a-5p expression levels further increased in CDTX + SCI muscle at D4 and D7 respectively (Fig. 2d). Overall, osteo-suppressive miRNAs response appears to be delayed overtime while osteogenic miRNAs response to muscle damage seems to persist longer in CDTX + SCI muscle compared to CDTX muscle (Fig. 2c, d).

SCI has previously been described to alter muscle microenvironment^17^, so we hypothesized that SCI could set the path to an altered, pathologic muscle microenvironment that favours osteo-chondrogenic differentiation. We studied the expression levels of myomiRs involved in myogenic progenitors’ (MPs) activation, proliferation, differentiation, or fusion. We observed a downregulation of miR-1-3p, miR-133a-3p, miR-206 and miR-378a-3p in muscle tissue 2 days after CDTX injury (Fig. 2e). Interestingly miR-1-3p was downregulated by SCI alone at D4 compared to SHAM surgery, while miR-206 was underexpressed in CDTX + SCI compared to CDTX alone 7 days after NHOs induction.

### SCI upregulates osteogenic markers expression and impairs osteo-suppressive miRNAs expression in FAPs after muscle injury

**Fig. 3:**
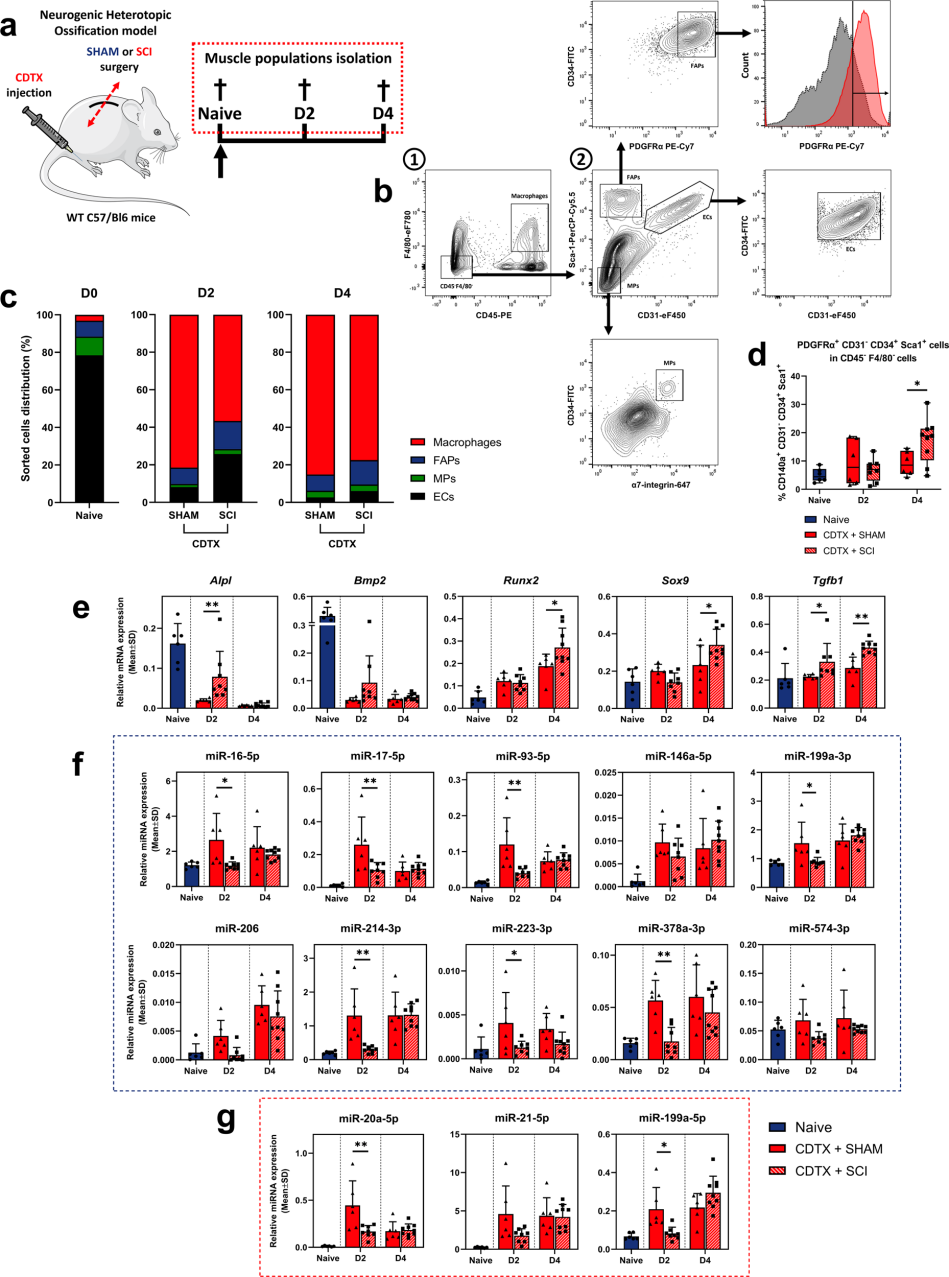
SCI upregulates osteogenic markers expression and impairs osteo-suppressive miRNAs expression in FAPs after muscle injury. **a** Experimental design of NHOs mouse muscle cellular subpopulations isolation in naive muscle and at day 2 and 4 after CDTX + SHAM or CDTX + SCI. This figure panel was partly generated using Servier Medical Art, provided by Servier, licensed under a Creative Commons Attribution 3.0 unported license. **b** Fibro-Adipogenic progenitors (FAPs), myogenic progenitors (MPs), macrophages and Endothelial Cells (EC) were sorted on the following strategy: macrophages were gated on CD45^+^ F4/80^+^ while non-hematopoietic lineage populations were all CD45^−^ F4/80^−^ as well as CD31^-^ Sca1^+^ CD34^+^ PDGFRα^+^ for FAPs, Sca1^−^ CD31^−^ CD34^low^ α7integrin^+^ for MPs and Sca1^+^ CD31^+^ CD34^+^ for ECs. **c** Muscle cell populations distribution on total sorted cells in naive muscle and CDTX + SHAM or CDTX + SCI muscle at day 2 and 4 (for each cell population, *n* = 6 in SHAM and naive groups, *n* = 8–9 in SCI groups). **d** Distribution of CD31^-^ Sca1^+^ CD34^+^ PDGFRα^+^ FAPs in CD45^−^ F4/80^−^ cells, in naive (*n* = 6), CDTX + SHAM (*n* = 6) and CDTX + SCI injured muscle (*n* = 8–9), 2 and 4 days after surgery. Relative **e** mRNA, **f** osteo-suppressive miRNAs and **g** osteogenic miRNAs expression levels measured by RT-qPCR from FAPs in naive, CDTX + SHAM and CDTX + SCI muscle at day 2 and 4 (*n* = 6–9). Histograms represent mean ± standard deviation (SD). Each dot represents independent biological samples from different mouse muscles (*n* = 6–9). Statistical differences were computed using two-way ANOVA with Šidák post hoc correction between CDTX + SHAM and CDTX + SCI groups (*P < 0.05, **P < 0.01, ***P < 0.001).

We previously described FAPs to be at the origin of NHOs development and sought to understand how they adopt an osteo-chondrogenic fate by describing osteogenic markers temporality and selected miRNAs^13^. We sorted major mononuclear cell populations from muscles after enzymatic digestion: FAPs (CD34^+^ CD31^−^ Sca1^+^ PDGFRα^+^ cells in CD45^−^ F4/80^−^ cells), MPs (α7Integrin^+^ CD34^low^ CD31^−^ Sca1^−^ cells in CD45^−^ F4/80^−^ cells), macrophages (CD45^+^ F4/80^+^ cells) and endothelial cells (ECs) (CD34^+^ CD31^+^ Sca1^+^ cells in CD45^−^ F4/80^−^ cells) (Fig. 3a, b). Because mineralization appeared as soon as D2 and osteogenic differentiation was already observed at D7, we focused on D2 and D4 post injury. As expected, cell populations distribution was severely disrupted following CDTX injury with fewer ECs, and a massive macrophages infiltration (Fig. 3c). Surprisingly, macrophages proportion was lower 2 days after CDTX + SCI compared to CDTX alone. Conversely, FAPs distribution in CDTX + SCI muscle was not different from acute injured muscle 2 days after injury, whereas FAPs proportion increased in NHO muscle after 4 days (Fig. 3d).

Then we measured the expression of osteo-chondrogenic mRNA in FAPs. We observed a drop in *Alpl* and *Bmp2* expression at D2 and D4 after CDTX injury compared to naive mice, however their expression levels remained higher in CDTX + SCI (Fig. 3e). *Runx2* and *Sox9* expression were activated by the CDTX injury with a significant overexpression in CDTX + SCI muscle at D4. Fibrosis marker *Tgfb1* was also more expressed in FAPs from CDTX + SCI muscle compared to CDTX alone both 2 and 4 days after NHOs induction (Fig. 3e). Osteo-suppressive miR-16-5p, miR-17-5p, miR-93-5p, miR-146a-5p, miR-199a-3p, miR-206, miR-214-3p, miR-223-3p, miR-378a-3p and miR-574-3p (Fig. 3f) and osteogenic miR-20a-5p, miR-21-5p, miR-199a-5p (Fig. 3g) expression levels were quantified and their expressions all followed a similar pattern, with increased expression at D2 in CDTX + SHAM group. Their expressions were also dampened in CDTX + SCI group, interestingly remaining close to naive FAPs expression levels (Fig. 3f, g). Collectively, these results suggest a miRNAs mediated osteo-suppressive mechanism activated in FAPs after CDTX injury, that FAPs fail to activate in CDTX + SCI muscle where NHOs occurs.

### Myogenic markers and miRNAs involved in muscle regeneration are downregulated by SCI in MPs from injured muscle

**Fig. 4:**
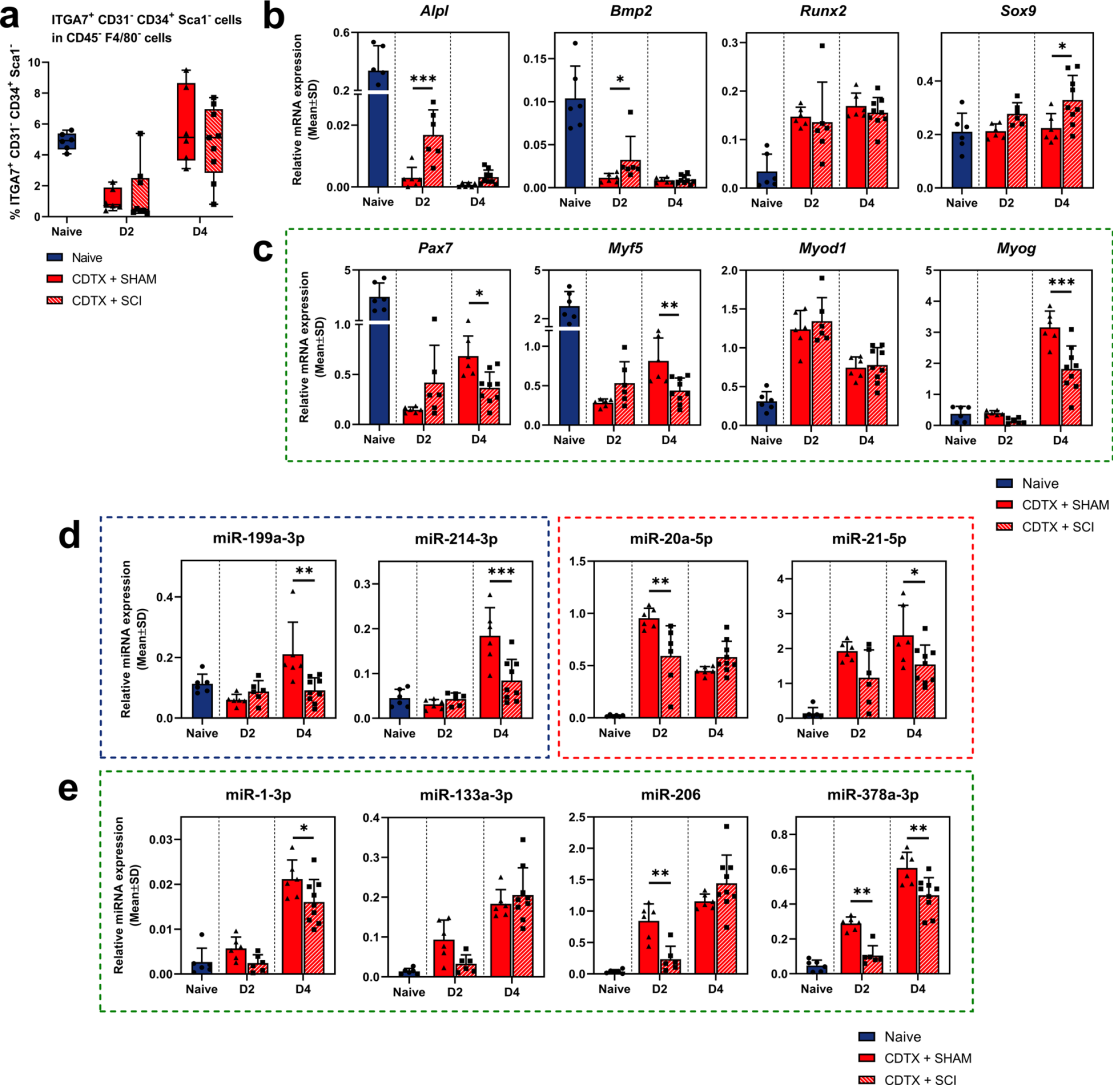
Myogenic markers and miRNAs involved in muscle regeneration are downregulated by SCI in myogenic progenitors from injured muscle. **a** Distribution of Sca1^−^ CD31^−^ CD34^low^ α7integrin^+^ MPs in CD45^−^ F4/80^−^ cells, in naive (*n* = 6), CDTX + SHAM (*n* = 6) and CDTX + SCI injured (*n* = 6–9) muscle. Relative **b** osteogenic markers mRNAs, **c** myogenic markers mRNAs, **d** osteo-suppressive/osteogenic miRNAs and **e** myomiRs expression levels measured by RT-qPCR from MPs in naive, CDTX + SHAM and CDTX + SCI muscle at days 2 and 4 (*n* = 6–9). Histograms represent mean ± standard deviation (SD). Each dot represents independent biological samples from different mouse muscles (*n* = 6–9). Statistical differences were computed using two-way ANOVA with Šidák post hoc correction between CDTX + SHAM and CDTX + SCI groups (*P < 0.05, **P < 0.01, ***P < 0.001).

While we recently reported that MPs were absent from mineralized nodules in NHOs 14 days post CDTX + SCI injuries, MPs remain a population of interest at the early stages of NHOs pathogenesis since SCI impairs muscle regeneration^13^. MPs cell number drastically decreased 2 days after CDTX injury before a rapid expansion at D4 (Fig. 4a). Interestingly, MPs expression of *Alpl* and *Bmp2* followed the same trend as FAPs with a lower expression in response to CDTX, partially alleviated in MPs from CDTX + SCI muscle (Fig. 4b). MPs also expressed osteo-chondrogenic markers, *Runx2* was expressed similarly in CDTX and CDTX + SCI groups compared to healthy muscle while *Sox9* expression was higher in CDTX + SCI MPs compared to MPs from CDTX-injured muscle at D4 (Fig. 4b). *Pax7* and *Myf5* are markers of MPs quiescence while *Myod1* is a marker of MPs activated state. As expected, we observed the activation of MPs in response to acute muscle damage in both CDTX and CDTX + SCI muscles, with a drop in *Pax7* and *Myf5* expression (Fig. 4c). Interestingly, *Pax7* and *Myf5* expression levels were lowered in MPs from CDTX + SCI muscle compared to CDTX muscle at D4 while *Myod1* expression levels increased steadily in both experimental conditions. Lastly, *Myog* is involved in myogenic differentiation. We found an activation at D4 in response to CDTX that was blunted in CDTX-SCI mice suggesting a delayed differentiation (Fig. 4c).

Osteo-suppressive miR-199a-3p and miR-214-3p expression levels were higher in MPs at D4 in CDTX group compared to CDTX + SCI and healthy muscle (Fig. 4d). Osteogenic miR-20a-5p and miR-21-5p expression levels were induced by CDTX at D2 and D4 while decreased in MPs from CDTX + SCI muscles, as found in FAPs (Fig. 4d). MiR-1-3p, miR-133a-3p, miR-206 and miR-378a-3p participate in various stages of MPs activation, proliferation, and myogenic differentiation. Their expression was induced after CDTX injury and was partially hindered in MPs from CDTX + SCI muscle at D2 and D4 (Fig. 4e). Taken together, these results indicate that MPs from CDTX + SCI muscle express less *Myog*, myomiRs and more osteogenic markers in the initial stages of ectopic mineralization development compared to CDTX damaged muscle only.

### Conditioned medium from LPS-stimulated-CD14^+^ macrophages (CM^+^) potentiates human FAPs osteogenic differentiation and increases osteo-chondrogenic markers and miRNAs expression

**Fig. 5:**
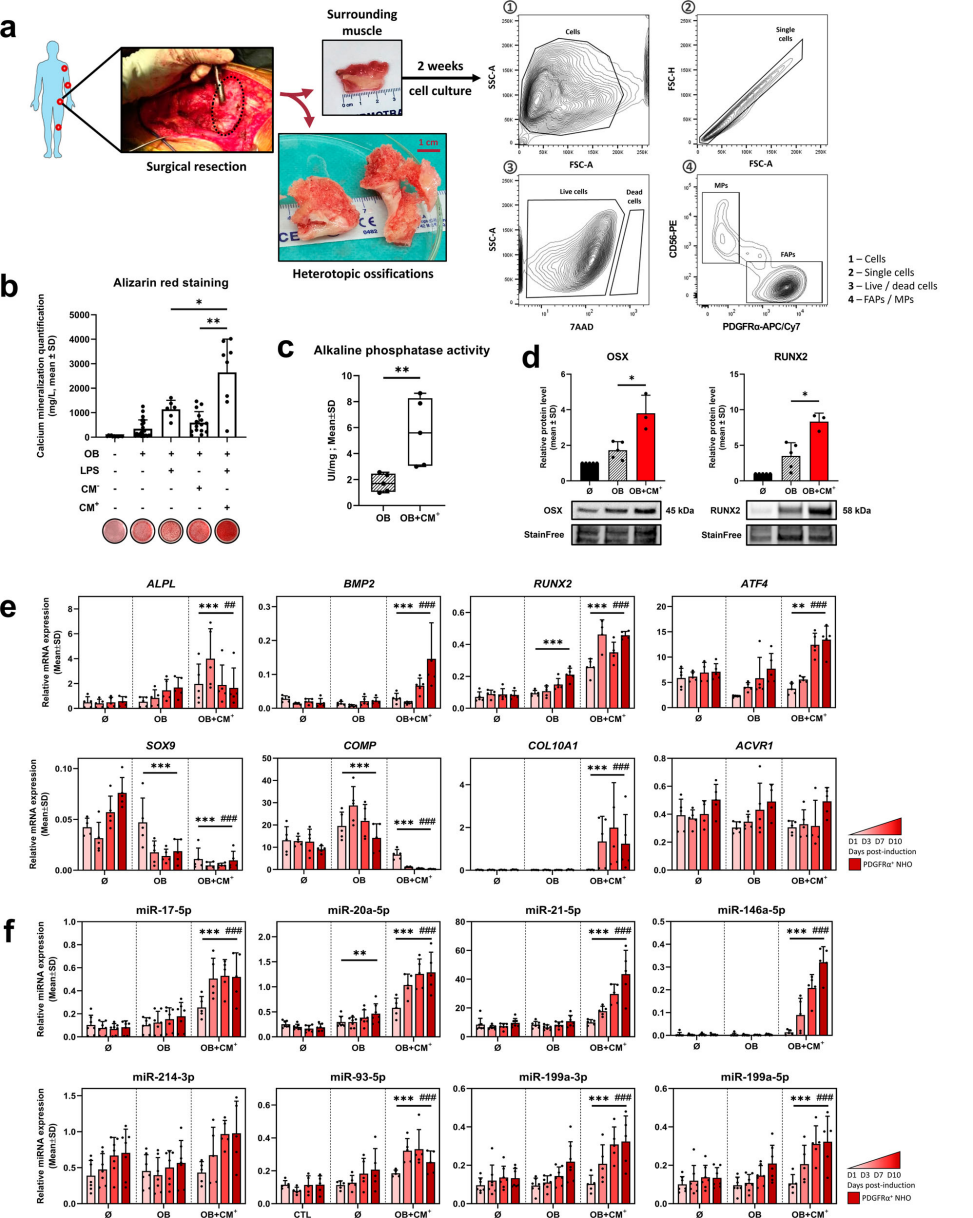
Conditioned medium from LPS-stimulated-CD14^+^ macrophages (CM^+^) potentiates human FAPs osteogenic differentiation and increases osteo-chondrogenic markers and miRNAs expression. **a** Experimental design of human muscle progenitors isolation from NHOs surgical resection. Briefly, human muscles were collected around the ectopic ossification, after surgical removal, mechanically and enzymatically minced, cultured for 2 weeks, and adherent cells were sorted on PDGFRα^+^ CD56^−^ for FAPs and PDGFRα^−^ CD56^+^ for MPs from 7-AAD^−^ live single events. **b** Osteogenic differentiation assay of human FAPs cultured in osteogenic conditions (OB), supplemented with lipopolysaccharides (LPS, 100 ng/mL) and naive (CM^−^) or LPS-stimulated conditioned medium (CM^+^) from macrophages, obtained through CD14^+^ isolation from human buffy coat, in a 1:10 ratio. Mineralization was observed using alizarin red staining and quantified at 405 nm after acid-SDS extraction. Each dot represents independent biological samples from different NHOs surgery (*n* = 6–18). **c** Alkaline phosphatase activity was assessed, 7 days after osteogenic induction with or without CM^+^ by monitoring the optical density at 405 nm after incubation with a commercial substrate at 37 °C (*n* = 5). **d** OSX and RUNX2 protein expression levels were quantified by western blot 7 days after osteogenic induction with or without CM^+^ (*n* = 3–5). Relative RT-qPCR **e** osteogenic mRNA and **f** miRNAs expression levels from FAPs control, OB, OB + CM^+^ at 1, 3, 7 and 10 days after osteogenic induction (*n* = 5) (*P < 0.05, **P < 0.01, ***P < 0.001 represent group effect compared to Ø condition ; ^#^*P* < 0.05, ^##^*P* < 0.01, ^###^*P* < 0.001 represent group effect compared to OB condition). Histograms represent mean ± standard deviation (SD). Each dot represents independent biological samples from different NHOs surgery. Statistical differences were calculated using Mann–Whitney *U* test for simple comparisons or two-way ANOVA with Tukey post hoc correction for multiple comparisons (*P < 0,05, **P < 0,01, ***P < 0,001).

To confirm our findings and explore their relevance to human NHOs, we used PDGFRα^+^CD56^−^ FAPs isolated from skeletal muscle surrounding NHOs surgical resection specimens (Fig. 5a). FAPs expressed classical mesenchymal stromal cells markers such as CD73, CD90 and CD105 as we previously described^13,36^. FAPs were cultured in osteogenic conditions (OB) with LPS, conditioned medium from naive (CM^−^) or LPS-stimulated CD14^+^ macrophages (CM^+^) to mimic the inflammatory environment. CM^+^ osteo-inducing potential and pro-inflammatory cytokines levels were described in previous work^8,27^ and we confirmed its ability to enhance FAPs mineralization (Fig. 5b) and alkaline phosphatase activity (Fig. 5c). OSX and RUNX2 protein expression were upregulated by the addition of CM^+^ compared to osteogenic differentiation medium alone (Fig. 5d). We next quantified osteo-chondrogenic markers 1, 3, 7 and 10 days post osteogenic induction (Fig. 5e). We observed that *ALPL*, *BMP2*, *RUNX2* and *ATF4* were upregulated by OB + CM^+^ compared to control growth medium or osteogenic differentiation medium alone, with increased expression overtime. Intriguingly, chondrogenic markers *SOX9* and *COMP* were downregulated by OB + CM^+^ whereas *COL10A1*, a marker of chondrocyte hypertrophy, was expressed only in OB + CM^+^ condition (Fig. 5e). Expression of *ACVR1*, a BMP receptor involved in *Fibrodysplasia Ossificans Progressiva* (FOP), the genetic form of HOs, was not altered by any experimental conditions (Fig. 5e).

We next explored the expression of previously identified miRNAs candidates from our NHOs mouse model (Supplementary Data 2). Osteo-suppressive miR-17-5p, miR-146a-5p, miR-93-5p and miR-199a-3p as well as osteogenic miR-20a-5p, miR-21-5p and miR-199a-5p were overexpressed in the OB + CM^+^ culture condition compared to control or osteogenic differentiation medium alone (Fig. 5f). Interestingly, osteo-suppressive miR-214-3p was not significantly overexpressed in OB + CM^+^ cultured FAPs (Fig. 5f). Hence, we confirmed that both osteo-suppressive and osteogenic miRNAs were upregulated in NHOs patients FAPs by a pro-inflammatory/pro-osteogenic environment, consistent with murine FAPs miRNAs response to CDTX injury.

### Synthetic miRNAs inhibitors and mimics transfections suppress both mineralization and osteo-chondrogenic markers in human FAPs

**Fig. 6:**
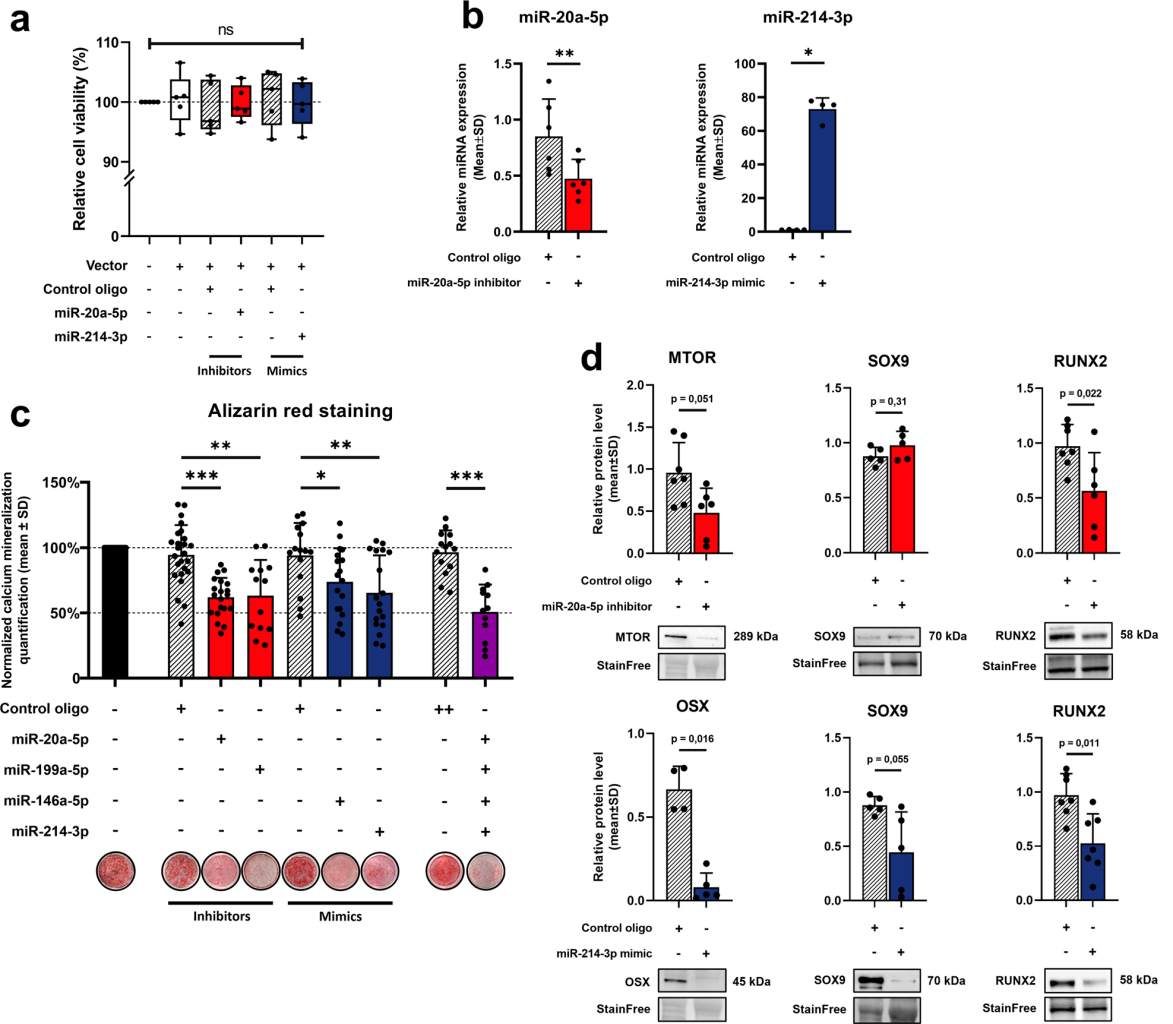
Synthetic miRNAs inhibitors and mimics transfections suppress both mineralization and osteo-chondrogenic markers expression in human FAPs. **a** FAPs viability was assessed by quantifying DAPI intake intensity by dead or apoptotic cells in flow cytometry in five experimental groups: control, lipofectamine RNAimax vector, vector+control oligo, vector+miR-20a-5p inhibitor and vector+miR-214-3p mimic. Inhibitors transfections were performed during 48 h at 10 nM and mimics at 1 nM for both control oligos and specific oligos (*n* = 5). **b** Relative RT-qPCR miRNAs expression levels in FAPs after control oligo or miR-20a-5p inhibitor and miR-214-3p mimic transfection (*n* = 5–6). **c** Osteogenic differentiation assays of transfected human FAPs cultured in osteogenic conditions (OB). Control inhibitor, miR-20a-5p inhibitor, miR-199a-5p inhibitors were used at 10 nM final and control mimic, miR-146a-5p mimic, miR-214-3p mimic at 1 nM final (*n* = 12–25). Values were normalized to OB condition alone. Statistical differences were calculated using unpaired *t* test with Welch correction (*P < 0,05, **P < 0,01, ***P < 0,001). **d** MTOR, SOX9 and RUNX2 protein expression levels from FAPs were quantified by western blot 7 days after osteogenic induction of control oligo or miR-20a-5p inhibitor transfection (top panel). OSX, SOX9 and RUNX2 protein expression levels were quantified by western blot 7 days after osteogenic induction of control oligo or miR-214-3p mimic transfected FAPs (bottom panel) (*n* = 5–7). Histograms represent mean ± standard deviation (SD). Each dot represents independent biological samples from different NHOs surgery. Unless stated otherwise, statistical differences were calculated using Mann–Whitney *U* test for simple comparisons or one-way ANOVA with Tukey post hoc correction for multiple comparisons (*P < 0.05).

To determine whether our miRNAs candidates contribute to FAPs osteogenic differentiation in NHOs, we transfected mimics or antimiR oligos in FAPs. Based on previous evidence, we selected two major candidates: the osteo-suppressive miR-214-3p and the osteogenic miR-20a-5p and two minor candidates: osteo-suppressive miR-146a-5p and osteogenic miR-199a-5p to perform functional analysis (Fig. 6). We ensured that neither the transfection vector nor specific inhibitors and mimics altered cell viability (Fig. 6a). As expected, miR-20a-5p inhibitor reduced miR-20a-5p expression and miR-214-3p mimics was abundant in transfected human FAPs (Fig. 6b). We quantified alizarin red staining after inducing osteogenic differentiation of transfected FAPs and observed that both the inhibition of osteogenic miR-20a-5p/miR-199a-5p and the overexpression of osteo-suppressive miR-214-3p/miR-146a-5p decreased human FAPs osteogenic mineralization (Fig. 6c). We also performed osteogenic differentiation assays in FAPs transfected with the 4 oligos and found a 50% decrease in mineralization compared to control (Fig. 6c). Finally, we studied protein expression of indirect but biologically relevant osteo-chondrogenic targets of miR-214-3p and miR-20a-5p (Fig. 6d). We found a decrease in MTOR and RUNX2 protein expression using miR-20a-5p inhibitor and a decrease in OSX, SOX9 and RUNX2 using miR-214-3p mimic in human FAPs (Fig. 6d). On the contrary, miR-214-3p analogs increased FAPs RUNX2 protein expression (Supplementary Fig. 3). Additionally, functional analysis of miR-17-5p inhibitor did not affect osteogenic properties of human FAPs, similarly to opposite oligos of precedingly described candidates, suggesting the involvement of saturation mechanisms and highlighting the importance of baseline expression levels (Supplementary Fig. 3).

## Discussion

Although it is currently admitted that NHOs follow an intramuscular osteochondral development, the involvement of the central nervous system injury in the initial physiopathological stage remains unclear. Our results uncover a link between muscle FAPs and MPs miRNAs expression and distant central nervous system injury leading to the onset of NHOs in a clinically relevant mouse model. After muscle injury, we found an increase in osteo-chondrogenic markers *Runx2*, *Sox9, Col10a1* and *Tgfb1*. Osteogenic and most osteo-suppressive miRNAs were activated in CDTX-injured muscle tissue while SCI increased the expression of the osteogenic miR-20a-5p in CDTX muscle at D4. Interestingly, the combination of muscle injury and SCI prompted osteogenic miRNAs response while partially inhibiting osteo-suppressive miRNAs expression. Among the most SCI-downregulated miRNAs 4 days after injury, we identified suppressors of osteogenesis and chondrogenesis : miR-214-3p, miR-199a-3p, miR-574-3p^37–40^. These results show that miRNAs landscape in skeletal muscle is profoundly altered in response to muscle damage and that SCI substantially modifies the osteogenic/osteo-suppressive miRNA balance.

Because miRNAs expression in whole muscle may reflect not only active regulations but also profound changes in cell composition, we next investigated miRNAs signature in sorted FAPs, the cells of origin of NHOs. We initially hypothesized that FAPs osteogenic miRNAs would be upregulated as observed in CDTX + SCI muscle, however, their expression levels were lowered at D2 compared to CDTX alone. Similarly, while osteo-suppressive miRNAs were upregulated in FAPs after injury, this response was blunted in CDTX-SCI group, including miR-214-3p, whereas osteo-chondrogenic markers were overexpressed. Interestingly, Eisner et al. have previously reported FAPs osteogenic phenotype during muscle inflammation in the onset of traumatic heterotopic ossifications^41^. These results suggest a miRNAs osteo-suppressive mechanism activated by FAPs in response to muscle injury and osteogenic signaling during muscle damage to prevent aberrant FAPs osteogenic commitment, which is impaired by SCI.

Our mouse model is characterized by a SCI-induced failure of muscle regeneration and ectopic ossification. To understand how SCI impairs muscle regeneration and induces NHOs, we next studied MPs. As MPs do not contribute to NHOs formation, we anticipated limited modulation of osteogenic and osteo-suppressive miRNAs^13^. Our results showed however that MPs osteo-suppressive miRNAs were downregulated 4 days after NHOs induction, as observed for FAPs miRNAs downregulation after 2 days. However, it should be noted that miR-214-3p has well-documented roles in myoblast proliferation and differentiation therefore the expression pattern we found may be totally disconnected from any osteogenic process^42,43^. Myogenic marker *Myog* and myomiRs were also downregulated in MPs from NHOs developing muscles suggesting altered or delayed myogenesis. Interestingly, SCI-downregulated myogenic miR-1-3p which targets the master regulator of chondrogenesis *Sox9*, found to be increased in MPs from NHO muscle^44^.

We next studied human FAPs sorted from patient’s muscle surrounding NHOs. We used our previously published model where FAPs are cultured with conditioned media from activated macrophages to mimic NHOs muscle environment in vitro^8,27^. We confirmed that the osteogenic differentiation and osteogenic markers expression were significantly increased. Interestingly, osteo-suppressive miR-214-3p was the only miRNA identified in both models that was not overexpressed in mineralizing conditions (CDTX + SCI and OB + CM^+^). The functional analysis revealed that miR-20a-5p and miR-199a-5p inhibitors as well as miR-146a-5p and miR-214-3p mimics reduced FAPs mineralization. Downstream osteo-chondrogenic markers were also decreased at protein level, including the key regulators RUNX2 and OSX. MiR-20a-5p and miR-199a-5p are involved in osteogenic differentiation of human stem cells, respectively regulating *BMP/SMAD* and *HIF1a* signaling^45–48^. On the other side of the spectrum, miR-214-3p and miR-146a-5p have both been described in osteo-suppressive mechanism by targeting *ATF4* and *SIRT1*^37,49^. It is interesting to note that miR-199a-3p and miR-199a-5p are the two arms of the same pre-miRNA but exhibit opposed roles on osteo-chondrogenic process, as well as different abundancy levels in mice FAPs. This suggests that the same pre-miRNA could contain both the stimulation and the inhibition signals, and that each strand has an independent fate after miRNA maturation. As such, investigating their active regulation such as sponging RNAs could be of great interest to decipher their role in NHOs pathogenesis. As transfection of a mix of the four candidates could not improve the osteogenic inhibition, we think that these miRNAs target similar osteogenic pathways in redundant ways. Taken together, our results suggest that FAPs osteogenic differentiation in NHOs is supported by blunted osteo-suppressive miRNAs response linked to SCI concomitant to osteogenic stimuli after muscle injury. Indeed, basal miRNAs expression levels prevent FAPs from undergoing osteogenic differentiation in healthy muscle or control medium, alike the simultaneous upregulation of both osteogenic and osteo-suppressive miRNAs in injured/regenerating muscle. Hence, overexpression of osteogenic markers and lack of osteo-suppressive miRNAs activation in differentiation and mineralization.

In the present study, we studied isolated FAPs (bone forming cells) and MPs (that fail regenerating) but not macrophages that are key players in NHOs. This is a limitation of our study, but also a deliberate choice, since the complexity of the results leads us to believe that a dedicated study is needed. Our results on whole muscle tissue bring clues and confirm the need for such a study. MiR-214-3p and miR-146a-5p have been reported to regulate M2 macrophage polarization^50,51^ while miR-20a-5p targets *TGFβ* signalling, favouring M1 pro-inflammatory macrophages phenotype^52,53^. As SCI exacerbates inflammation, those miRNAs could represent promising targets to couple osteo-suppressive effect and pro-resolution macrophages capacities^54^. Macrophages differentiation and polarization may be studied to better understand how they affect NHOs development and how the SCI could potentially skew miRNAs signaling leading to altered MΦ/M1/M2 activations. The question of a transfer of miRNAs from macrophages to FAPs that could support their osteoblastic differentiation goes beyond the aim of the present study, but further investigations may be of great interest since inflammation is a key mechanism in NHOs. Of further note, in a rat model of traumatic brain injury, injured neurons produced and released osteogenic miRNAs that accelerate bone healing^55^. It should however be acknowledged that despite abundant literature describing miRNAs signaling between cells, the importance of such mechanisms is still debated^56–58^. Taken together, our results show for the first time that central nervous system injury substantially modifies the miRNAs landscape in injured muscle as well as in FAPs and MPs. The functional analysis in NHO patient’s cells demonstrates that several dysregulated miRNAs alter FAPs osteogenic differentiation. Our results suggest that NHOs pathogenesis is not regulated by a single miRNA but rather by a finely tuned balance between osteogenic and osteo-suppressive miRNAs. Muscle damage upregulates both osteogenic and osteo-suppressive miRNAs while central nervous system damage disrupts this equilibrium by lowering osteo-suppressive miRNAs response. More work is needed to determine whether intercellular miRNAs signaling also contributes to NHOs pathogenesis and how macrophages participate in this complex signaling.

## Methods

### NHOs mouse model

Animal experiments were conducted in accordance with the Directive 2010/63/EU of the European Parliament and of the Council of September 22, 2010 (2010/63/EU). The experimental procedures were approved by the “C2EA-26” Ethics committee in accordance with French Ministry of Research regulations (#25901-2020060515467494).

Six to eight weeks old female C57Bl/6 mice from Janvier Labs (France) underwent spinal cord transection injury (SCI) between T11-T13 or SHAM surgery (control) followed by intramuscular injections of 12 µM cardiotoxin (CDTX, L8102, Latoxan, left hindlimb) and phosphate-buffered saline (PBS, right hindlimb) into each gastrocnemius (150 µL). Mice were euthanized at 0 (naive), 2, 4 or 7 days post-surgery and both gastrocnemius were harvested and either processed for cell isolation or flash frozen in isopentane (M32631, Sigma-Aldrich) and embedded in OCT. Naive mice were also used. The occurrence and volume of NHOs in male or female mice is not different in our model, female mice were used to because urination is blocked by the SCI and triggering it much easier in females, thereby reducing anesthesia duration and infection risk.

### Muscle histology and immunohistochemistry

In all, 8 µm cryosections were dried and stained with Hematoxylin Phloxine Saffron (HPS) (Dako). For Von Kossa staining, cryosections were incubated 30 min at RT in 1% silver nitrate in the dark followed by 15 min incubation in UV chamber and nuclear fast red counterstain. All sections were analyzed using a Pannoramic Midi II slide scanner and Case Viewer software (3D HISTECH Ltd.).

For immunohistochemical staining, cryosections were fixed in 4% PFA 15 min, incubated in PBS-Triton 0.5% 15 min followed by 1 h in PBS-BSA 1% at RT. Muscle sections were incubated with rabbit polyclonal anti-laminin antibody (dilution 1/200, Abcam) or rabbit polyclonal anti-osteocalcin or anti-Collagen III antibody (dilution 1/100 and 1/200, Abcam) in PBS-BSA 1% overnight at 4 °C. Corresponding normal polyclonal rabbit IgG was used as negative control (Abcam). Sections were washed with PBS and incubated with secondary antibody Donkey anti-rabbit Alexa Fluor 594 or Goat anti-rabbit Alexa Fluor 488 (1/500, Thermo) in PBS-BSA 1% for 1 h à room temperature. Slides were finally mounted in Vectashield antifade mounting medium with DAPI (Vector). All sections were analyzed using inverted epifluorescence microscope (DMi8, Leica) and Fiji software (ImageJ)

### Murine FAPs, macrophages and MPs isolation

Cellular isolations were performed following an adapted protocol from ref. ^59^. Briefly, gastrocnemius muscles were mechanically minced and incubated in 10 mg/mL collagenase B (11 088 831 001, Roche) with 2.4 U/mL Dispase II (17101015, Gibco) at 37 °C for 30 min. Digestion was stopped using α-MEM 50% FBS solution and filtered through a 70 µm cell strainer. Red blood cell lysis was performed using commercial buffer (130-094-183, Miltenyi). Cells were incubated in FcBlock (130-092-575, Miltenyi) for 30 min and control isotypes and antibodies were added at the indicated concentrations (see antibody table). After incubation, cells were washed and filtered through a 30 µm cell strainer before sorting. After sorting (Fig. 3a), cells were collected in 700 µL QIAzol lysis reagent (79306, Qiagen) to perform microRNAs and mRNAs screening.

### microRNAs screening and RT-qPCR analysis

Total RNAs were extracted from NHOs mice gastrocnemius biopsy or from FAPs cultured at 8000 cells/cm^2^ using QIAzol lysis reagent (79306, Qiagen) and chloroform (C2432, Sigma-Aldrich) extraction. RNA precipitation was performed using isopropanol (34965, Sigma-Aldrich) and GlycoBlue (AM9515, Ambion) for 20 min at −20 °C. Pellet were washed with 75% EtOH (20824.321, BDH Chemicals) before RNAse free water resuspension. Total RNA concentration were evaluated by NanoDrop (ThermoFisher), pre-diluted to 20 ng/µL for mRNA or 5 ng/µL for microRNA and stored at −80 °C. Reverse transcriptions were performed with RT² First Strand kit (330404, Qiagen) for mRNA and miRCURY LNA RT kit (339340, Qiagen) for microRNA following manufacturer instructions. Post-RT cDNA were respectively diluted to 1:80 (mRNA) and 1:20 (microRNA) before RT-qPCR amplification using RT² SYBR Green (330502, Qiagen) and miRCURY LNA SYBR Green (339345, Qiagen). NHOs mice microRNAs screening was performed using miRNome Mouse&Rat panel I + II (V5, 339322, Qiagen). These panels contain miRNAs that are highly expressed and more likely to be differentially expressed in diseases based on how often they are cited in the literature. The quantification cycle was determined as the second derivative maximum by the LightCycler software. In each experiment, the three most stable reference genes or microRNAs were selected using Genorm software (v3.4). The expression of the target genes/miRNAs was quantified using 2^ΔCt^ method (ΔCt = Ct_reference_ − Ct_target_) with each reference. The final quantified value is the geometric mean of the 3 values obtained. For in vivo screening, the most stable genes and miRNAs were *Hprt*, *Rplp0*, *Ppia* and hsa-miR-24-3p, hsa-miR-23b-3p, hsa-miR-125b-5p. For in vitro experiments *FGFR1*, *ITGA1*, *BGN* and hsa-miR-24-3p, hsa-miR-103a-3p, hsa-miR-143-3p were selected as references.

### Human ethics statement

All human samples were obtained with the informed consent of the patients, the approval from the people protection committee (CPP approval n°09025, study BANKHO) and the approval from the National Commission for Informatics and Liberties (CNIL approval n°Eyo1066211J).

### Human PDGFRα^+^CD56^−^ FAPs isolation

Muscle surrounding NHOs were collected from NHOs surgical wastes following their excision from patients with brain injuries, spinal cord injuries or strokes. NHOs resection surgeries were performed at Raymond Poincaré Hospital (Garches, France). Muscle fragments were mechanically minced, placed in a 50 ml Falcon tube, and incubated in 1.5 mg/ml pronase (#10165921001, Sigma-Aldrich) in α-MEM, 45 min in a 37 °C water bath. After addition of α-MEM supplemented with 15% FBS and 1% P/S antibiotics, the cell suspension was filtered through a 100 µm cell strainer followed by a 40 µm cell strainer. Isolated muscle cells were maintained in α-MEM supplemented with 15% FBS, 1% P/S antibiotics and 10 ng/ml basic fibroblast growth factor (bFGF, 233-FB, R&D Systems) for 2 weeks. Human muscle progenitor cells were trypsinized and incubated 30 min with biotinylated anti-human PDGFRα (2217560, Sony) and CD56-PE (clone B159, BD Pharmigen) monoclonal antibodies in PBS 2% FCS, 2 mM EDTA or with control isotypes IgG1 PE (A07796, Beckman Coulter) and IgG1 APC/Cy7 (AB-105-C, R&D Systems). Cells were washed and incubated 30 min with Streptavidin-APC/Cy7 (262040, ICyt) and the viability dye 7-AAD (2702020, Sony). Cells were washed and filtered through a 30 µm cell strainer (Sysmex) and sorted using a FACSAria III SORP sorter (BD Biosciences). PDGFRα^+^CD56^−^ FAPs were seeded at 3000 cells per cm^2^ in α-MEM supplemented with 10% FBS and 1% P/S antibiotics.

### Human CD14^+^ monocytes isolation and preparation of conditioned medium (CM^+^)

Human CD14^+^ blood monocytes were isolated from whole blood donation buffy coat from the Centre de Transfusion Sanguine des Armées (CTSA, Clamart, France). Peripheral blood mononuclear cells (PBMC) were recovered from using Ficoll gradient (Pancoll, Pan-Biotech). CD14^+^ monocytes were then isolated following magnetic separation using CD14 microbeads (130-050-201, Miltenyi Biotec). CD14^+^ cells were seeded at 1.25 × 10^6^ cells per cm^2^ and stimulated with lipopolysaccharides (LPS) (CM^+^, 100 ng/mL, L6529, Sigma-Aldrich) or naive without LPS (CM^−^) for 72 h. Conditioned medium were collected, centrifuged 5 min at 500× *g*, and pooled CM^+^ were used in a 1 to 10 ratio in cell culture medium during osteogenic differentiation assays.

### In vitro osteogenic differentiation assay and mineralization quantification

Human PDGFRα^+^CD56^−^ FAPs were seeded at 7900 cells/cm^2^ in α-MEM 10% FBS 1% P/S. After 3 days, culture medium was replaced by control medium (α-MEM 10% FBS 1% P/S antibiotics) or osteogenic medium (α-MEM 10% FBS 1% P/S antibiotics, 52 µg/mL dexamethasone, 12.8 µg/mL ascorbic acid and 2.15 mg/mL β-glycerophosphate (D-1159, A-8960, G-9422, Sigma-Aldrich) for 13 days. At the end of the differentiation process, cells were washed in PBS, fixed in 70% ethanol, washed in water, and stained in 20 g/L Alizarin red S (A5533, Sigma-Aldrich). Excess stain was removed during 3 washing steps. Alizarin Red S dye was extracted with 0.5 N hydrochloric acid, 5% sodium dodecyl sulfate (SDS) and quantified by spectrophotometry at 405 nm.

### Alkaline phosphatase activity assay

Alkaline phosphatase activity assays (AP0100, Sigma) were realized according to manufacturer’s instructions.

### AntimiR and mimic transfection

Functional analyses were performed using miRCURY LNA Power Inhibitors (10 nM final, 339121, Qiagen) and miRCURY LNA Mimics (1 nM final, 339173, Qiagen). Oligonucleotides were incubated 5 min at room temperature with Lipofectamine RNAiMAX (1.5 µL/mL, 13778075, ThermoFisher) in α-MEM prior to cell supplementation. After 48 h, culture medium was changed for osteogenic differentiation medium (see before). Viability was assessed by measuring dapi intensity in flow cytometry. We also performed negative control western blots to avoid the case of RISC complex saturation by analogous sequences leading to non-specific miRNAs biogenesis dysregulation as described by Khan et al.^60^.

### Viability assay

To assess viability of human FAPs after transfection with miRNAs inhibitors or mimics, we stained cells after 48 h of transfection with 5 µg/mL of DAPI for 5 min. Fluorescence was quantified in flow cytometry (CytoFLEX, Beckman Coulter).

### Western blot

Human PDGFRα^+^CD56^−^ FAPs in osteogenic assays were PBS washed before cell lysis incubation using PBS, Triton X-100 1% NP40 1%, SDS 0.1% deoxycholic acid 0.5% and protease inhibitors (11697498001, Roche) buffer. Cells were scrapped and incubated 20 min on ice. Supernatants were collected after centrifugation (13,000× *g*, 15 min at 4 °C). Protein concentrations were quantified using Pierce BCA protein assay kit (23252, ThermoFisher Scientific) following the manufacturer’s instructions. In total, 20 µg protein were loaded for electrophoresis in 10% acrylamide SDS-PAGE gels (161-0182, BioRad) before transfer on PVDF membrane (Trans-Blot Turbo, BioRad). Membrane saturations were performed for 2 h using 5% non-fat dry milk and subsequently incubated with specific antibodies (see antibody table) at 4 °C overnight. Membranes were probed with HRP secondary antibody (1:200,000, ab205718, Abcam) at room temperature for 2 h. After Enhanced ChemiLuminescence incubation (ECL, 170–5061, BioRad), imaging and densitometric analysis were performed on a ChemiDoc XRS+ (BioRad) using ImageLab for whole membrane StainFree normalization. Uncropped blots with molecular weights are presented in Supplementary Fig. 4.

### Statistics and reproducibility

All graphs and dots represent independent biological samples from different animals, including sorted muscle progenitors populations, or from different human biopsies. Statistical analyses were performed using GraphPad Prism (9.1.1). All plots represent mean ± standard deviation (SD) of different biological samples. Statistical differences were calculated using non-parametric two-sided Mann–Whitney *U* test for simple comparisons, one-way ANOVA with Tukey post hoc correction for multiple comparisons and two-way ANOVA with Tukey post hoc correction for multiple intra-groups comparisons or Šidák post hoc correction for multiple inter-groups comparisons (one-sided) (*P < 0.05, **P < 0.01, ***P < 0.001).

#### Supplementary reagents

Antibody concentrations and miRNAs sequences are provided in separate tables.

### Reporting summary

Further information on research design is available in the Nature Portfolio Reporting Summary linked to this article.

### Supplementary information

Supplementary information

Description of Additional Supplementary Material

Supplementary Data 1

Supplementary Data 2

Reporting Summary

## Data Availability

The authors declare that all data supporting the findings of this manuscript are available within the paper figures and supplementary figures. Individual numerical values can be found in “Supplementary Data 1” file. The RT-PCR profiling data discussed in this publication have been deposited in NCBI’s Gene Expression Omnibus^61^ and are accessible through GEO Series accession number GSE241333. Uncropped blots with molecular weights are presented in Supplementary Fig. 4.
