# Supplementary material for: Spinal cord injury dysregulates fibro-adipogenic progenitors miRNAs signaling to promote neurogenic heterotopic ossifications
Source: Commun Biol. 2023 Sep 12;6:932. doi: 10.1038/s42003-023-05316-w (PMC10497574; doi:10.1038/s42003-023-05316-w)

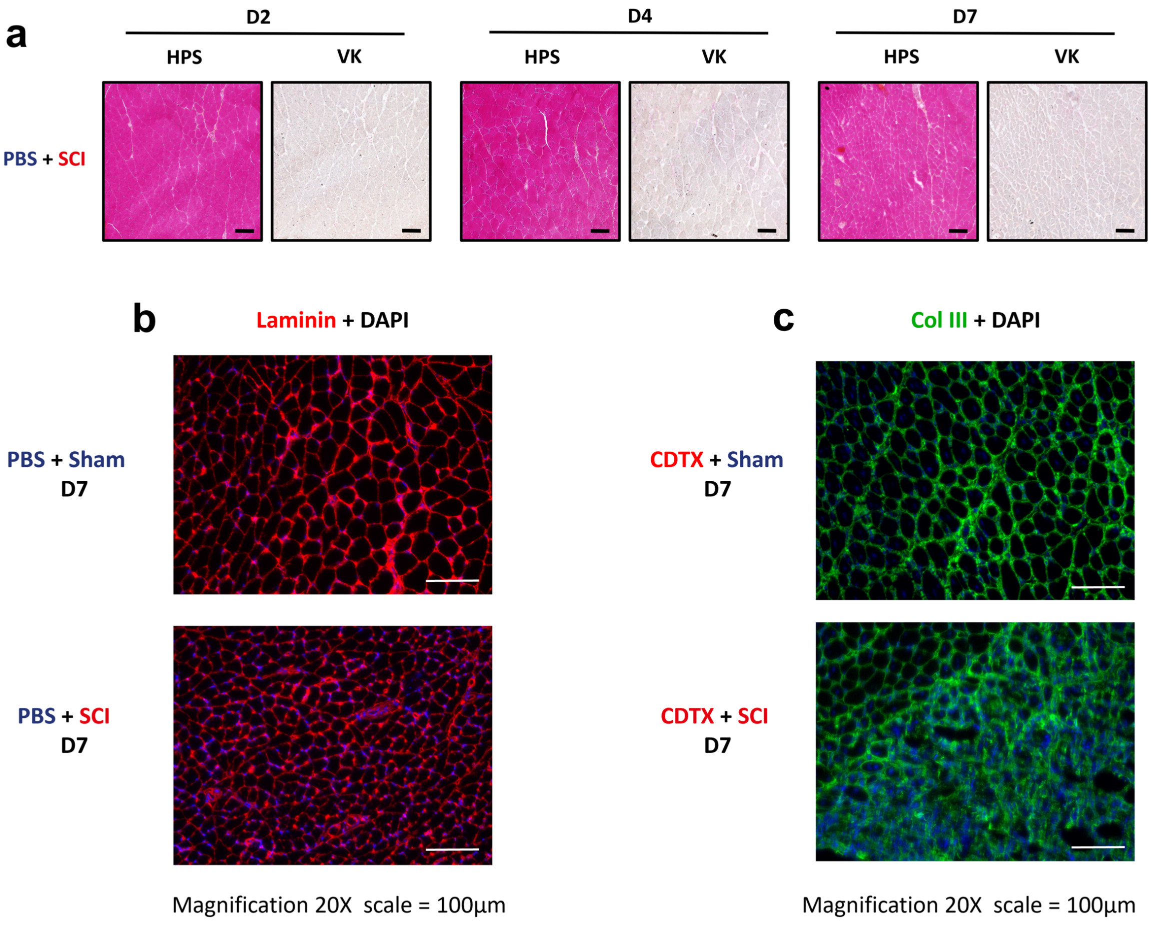

Supplementary Figure 1:

(a) Transversal cryosections of PBS+SCI gastrocnemius muscle stained with Hematoxylin Phloxine Saffron (HPS) or Von Kossa (VK) counterstained with nuclear fast red at day 2, 4 and 7. (magnification 10X, scale bar = 100 µm).
Laminin **(b)**, magnification 20X; scale bar = 100 µm, and collagen III **(c)**, magnification 10X; scale bar = 100 µm, immunostaining with DAPI co-staining on PBS+SHAM / PBS+SCI or CDTX+SHAM / CDTX+SCI gastrocnemius transversal cryosections 7 days after injury.

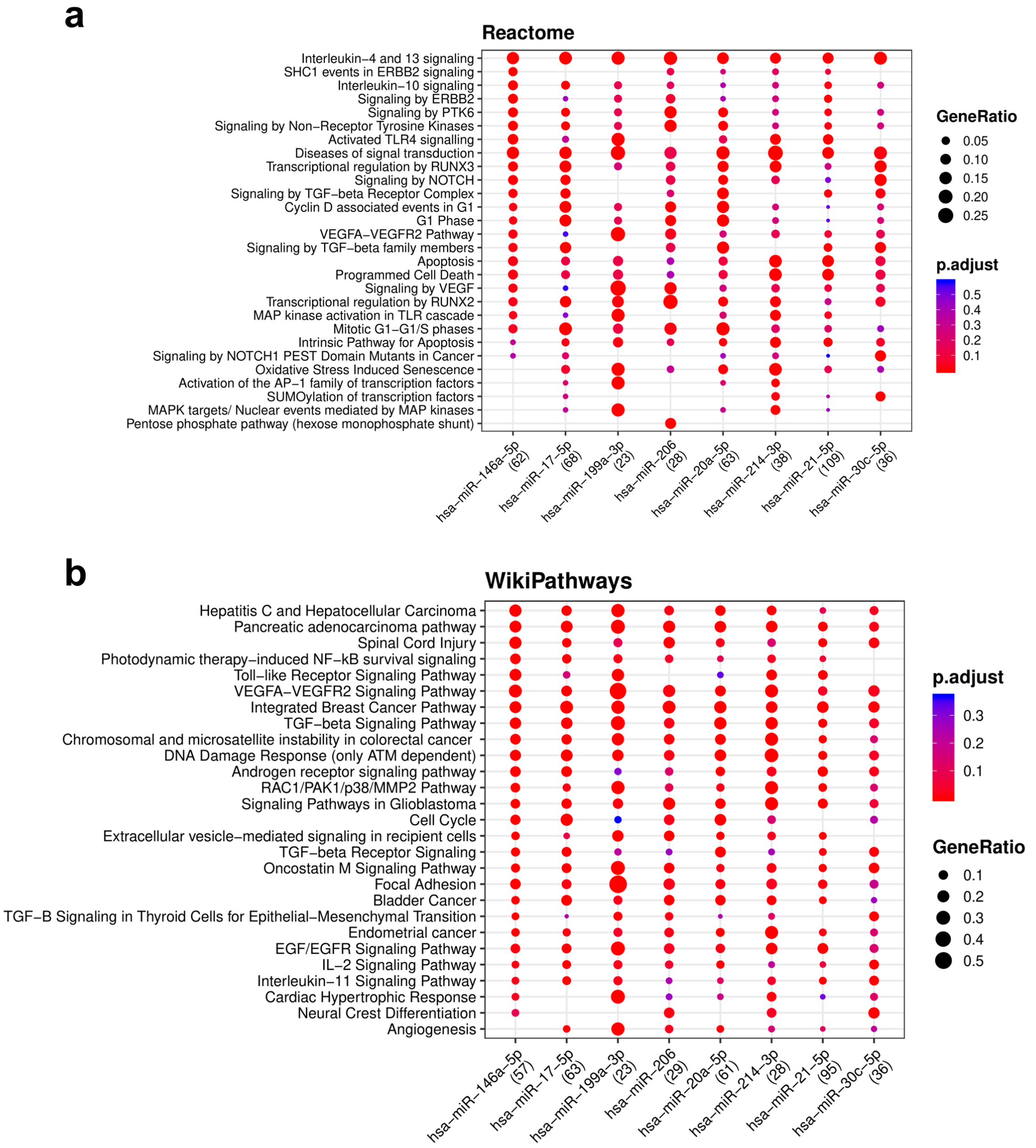

Supplementary Figure 2:

Reactome **(a)** and WikiPathways **(b)** database enrichment analysis of most up-regulated and down-regulated miRNAs

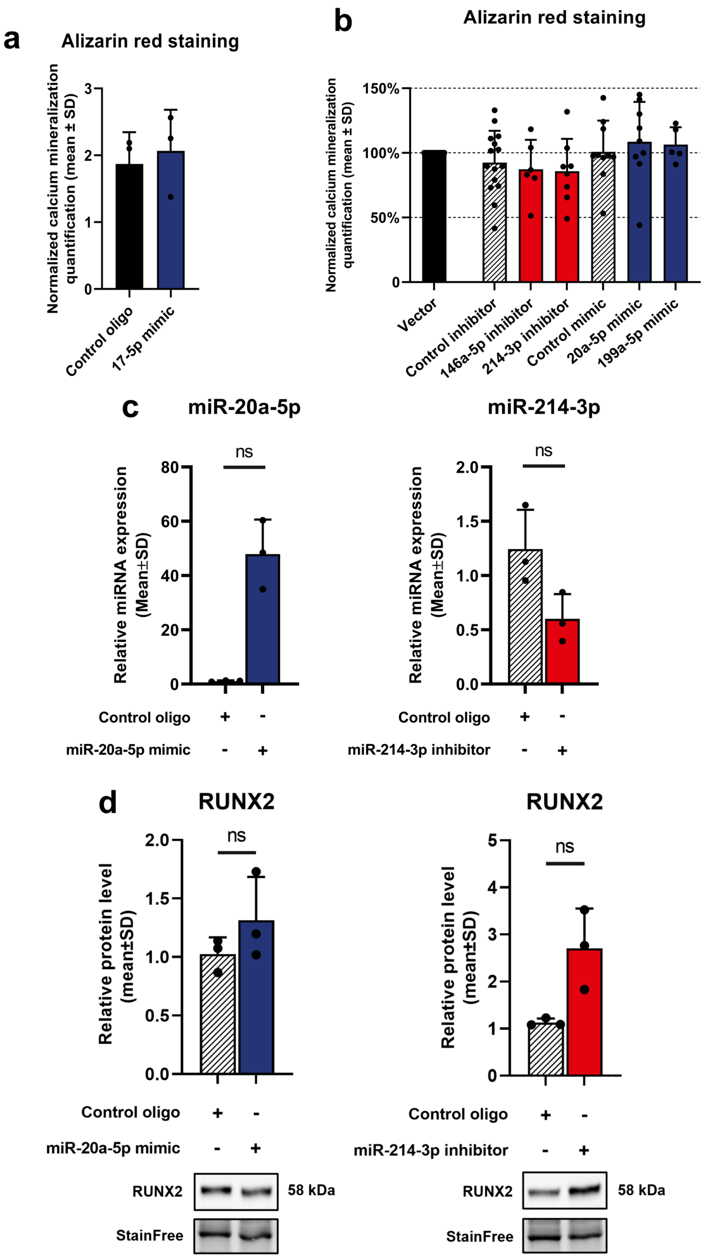

Supplementary Figure 3:

Osteogenic differentiation assays of transfected human FAPs cultured in osteogenic conditions (OB) (**a** & **b**). Inhibitors transfections were performed during 48h prior to osteogenic induction at 10 nM and mimics at 1 nM for both control oligos and specific oligos (n=3-15). **(c)** Relative RTqPCR miRNAs expression levels in FAPs after control oligo or miR-20a-5p mimic and miR-214-3p inhibitor transfection (n=3). **(d)** RUNX2 protein expression levels from FAPs were quantified by Western blot 7 days after osteogenic induction of control oligo, miR-20a-5p mimic or miR-214-3p inhibitor transfection (n=3). Histograms represent mean ± standard deviation (SD). Each dot represents independent biological samples from different NHOs surgery. Statistical differences were calculated using Mann-Whitney U test for simple comparisons.

Supplementary Figure 4: Uncropped blot images

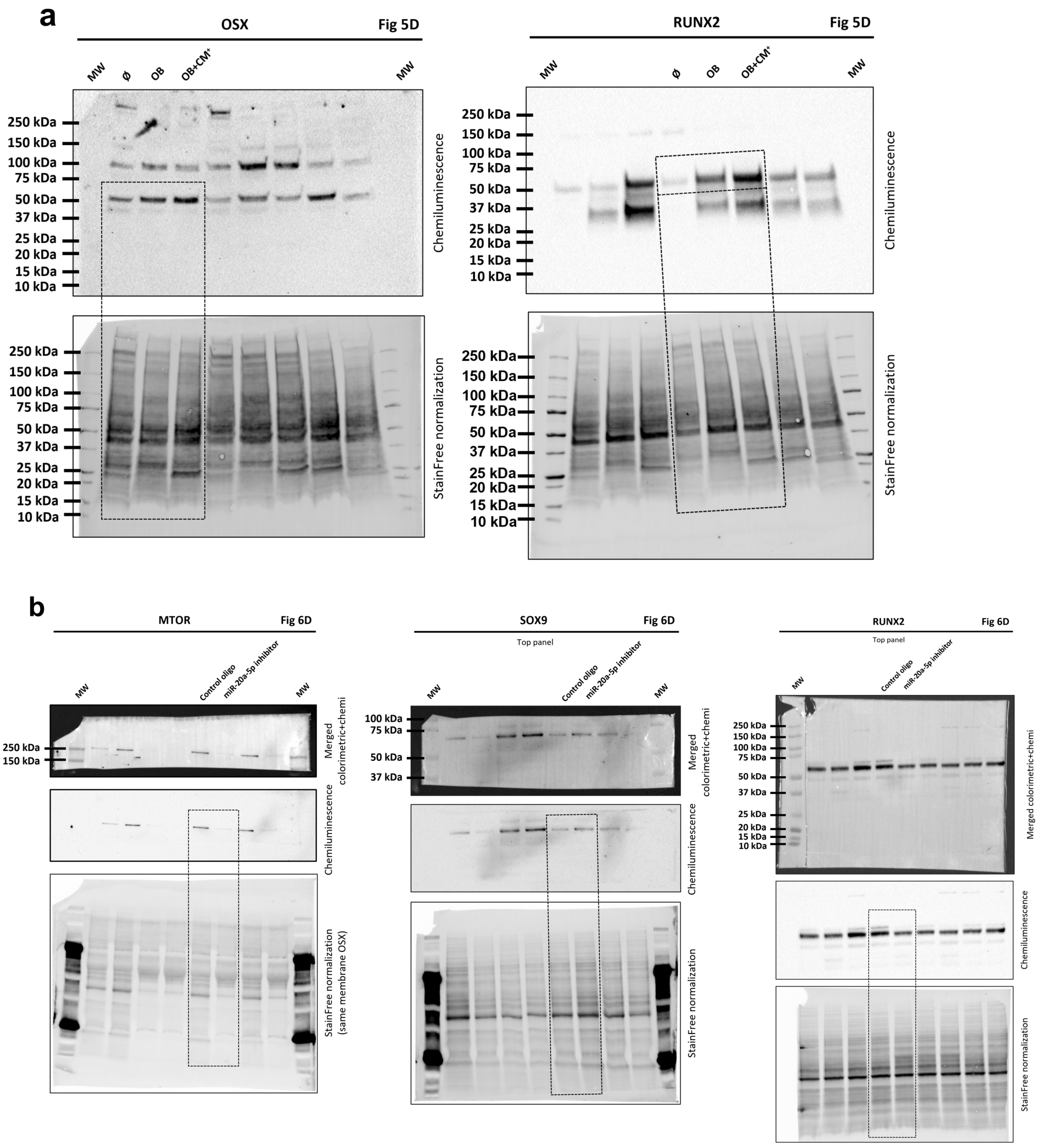

Supplementary Figure 4, continued

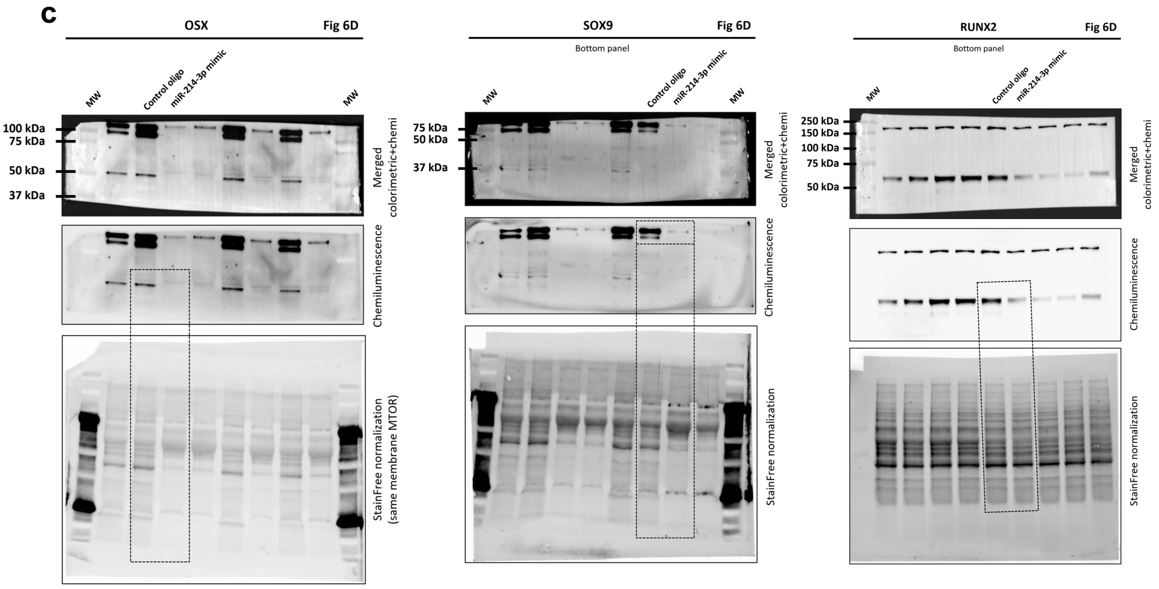

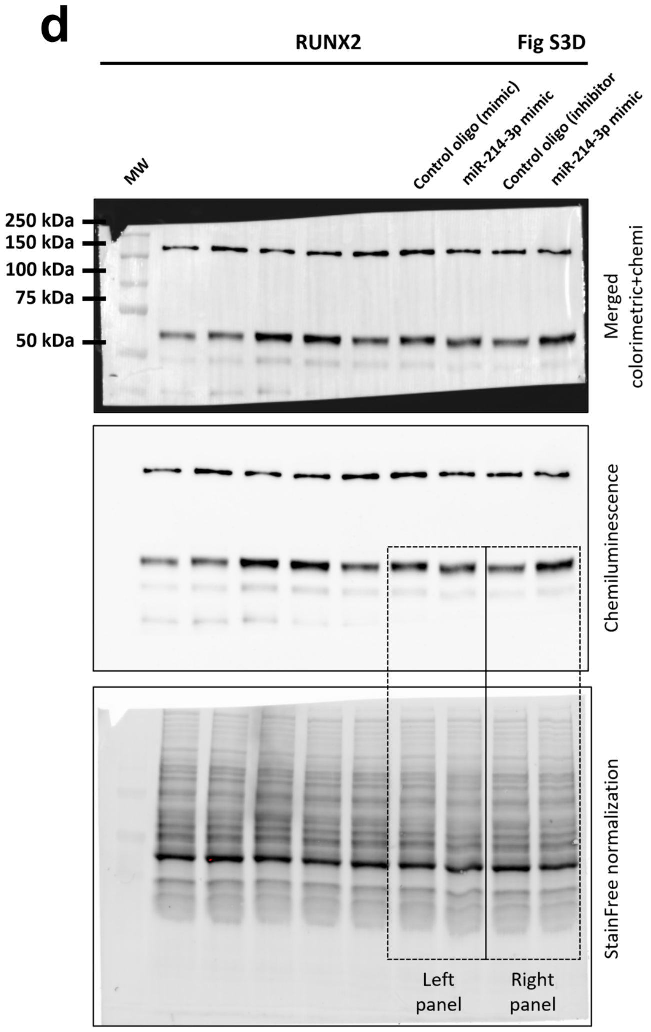

Supplement: Supplementary file 1 — Supplementary information [file 42003_2023_5316_MOESM1_ESM.pdf]
