# Supplementary material for: Spinal cord injury dysregulates fibro-adipogenic progenitors miRNAs signaling to promote neurogenic heterotopic ossifications
Source: Commun Biol. 2023 Sep 12;6:932. doi: 10.1038/s42003-023-05316-w (PMC10497574; doi:10.1038/s42003-023-05316-w)
Supplement: Supplementary file 2 — Description of Additional Supplementary Material [file 42003_2023_5316_MOESM2_ESM.docx]

**Description of Additional Supplementary Files**

**File name:** Supplementary Data 1

**Description:** This file consists in an excel file recapitulating all individual numerical data obtained in the experiment, that are presented in the figures as well as in the supplementary figures

**File name:** Supplementary Data 2

**Description:** This file consists in an excel file recapitulating information on the oligonucleotides and antibodies used in the experiment.
