# Supplementary material for: Spinal cord injury dysregulates fibro-adipogenic progenitors miRNAs signaling to promote neurogenic heterotopic ossifications
Source: Commun Biol. 2023 Sep 12;6:932. doi: 10.1038/s42003-023-05316-w (PMC10497574; doi:10.1038/s42003-023-05316-w)
Supplement: Supplementary file 5 — Reporting Summary [file 42003_2023_5316_MOESM5_ESM.pdf]

nature portfolio

Corresponding author(s): COMMSBIO-22-3872A
Sebastien BANZET

Last updated by author(s): Aug 25, 2023

Reporting Summary

Nature Portfolio wishes to improve the reproducibility of the work that we publish. This form provides structure for consistency and transparency in reporting. For further information on Nature Portfolio policies, see our Editorial Policies and the Editorial Policy Checklist.

Statistics

For all statistical analyses, confirm that the following items are present in the figure legend, table legend, main text, or Methods section.

n/a	Confirmed	
☐	☒	The exact sample size (n) for each experimental group/condition, given as a discrete number and unit of measurement
☐	☒	A statement on whether measurements were taken from distinct samples or whether the same sample was measured repeatedly
☐	☒	The statistical test(s) used AND whether they are one- or two-sided<br>*Only common tests should be described solely by name; describe more complex techniques in the Methods section.*
☒	☐	A description of all covariates tested
☐	☒	A description of any assumptions or corrections, such as tests of normality and adjustment for multiple comparisons
☐	☒	A full description of the statistical parameters including central tendency (e.g. means) or other basic estimates (e.g. regression coefficient) AND variation (e.g. standard deviation) or associated estimates of uncertainty (e.g. confidence intervals)
☐	☒	For null hypothesis testing, the test statistic (e.g. F, t, r) with confidence intervals, effect sizes, degrees of freedom and P value noted<br>*Give P values as exact values whenever suitable.*
☒	☐	For Bayesian analysis, information on the choice of priors and Markov chain Monte Carlo settings
☒	☐	For hierarchical and complex designs, identification of the appropriate level for tests and full reporting of outcomes
☒	☐	Estimates of effect sizes (e.g. Cohen's d, Pearson's r), indicating how they were calculated

Our web collection on statistics for biologists contains articles on many of the points above.

Software and code

Policy information about availability of computer code

Data collection	Histology : Pannoramic Viewer software for data collection on 3D HISTECH Pannoramic Scan II digital slide scanner.<br>Immunohistochemistry : Leica Application Suite LAS software for data collection on epifluorescence microscope DMi8 (Leica)<br><br>Flow cytometry : BD FACSDiva 7.0 software for data collection on BD Biosciences' FACSAria III SORP (cell sorting). CytExpert 2.3 software for data collection on Beckman Coulter's CytoFLEX (cell phenotyping).<br><br>RTqPCR : LightCycler 480 1.5.1 software for data collection on Roche's LightCycler480 II.<br><br>Western Blots : ChemiDoc Imaging System integrated software.
Data analysis	FlowJo v10.6.2 : Flow cytometry analysis software; GraphPad Prism 8 : Analysis and graphing software, GeNorm v3.4, Excel 2016, Mienturnet; CaseViewer: Analysis of histology; Fiji: IHC staining; ImageLab : quantification and normalization of WesternBlot.

For manuscripts utilizing custom algorithms or software that are central to the research but not yet described in published literature, software must be made available to editors and reviewers. We strongly encourage code deposition in a community repository (e.g. GitHub). See the Nature Portfolio guidelines for submitting code & software for further information.

Data

Policy information about availability of data

All manuscripts must include a data availability statement. This statement should provide the following information, where applicable:

- Accession codes, unique identifiers, or web links for publicly available datasets
- A description of any restrictions on data availability
- For clinical datasets or third party data, please ensure that the statement adheres to our policy

All dataset can be provided if necessary or requested after initial submission in a Supplementary Source Data File.

Human research participants

Policy information about studies involving human research participants and Sex and Gender in Research.

Reporting on sex and gender	Findings apply to both sexes.
Population characteristics	The population is constituted of men and women from 26 to 72 years old suffering from NHO following traumatic brain injury, spinal cord injury or stroke.
Recruitment	Participants were recruited before NHO surgical resection and informed on the becoming of surgical wastes, on which they fully agreed.<br>Tissue residus were collected during surgical resection of pathological NHO of the hip.
Ethics oversight	All human samples were obtained with the informed consent of the patients, the approval from the people protection committee (CPP approval n°09025, study BANKHO) and the approval from the National Commission for Informatics and Liberties (CNIL approval n°Eyo1066211J).

Note that full information on the approval of the study protocol must also be provided in the manuscript.

Field-specific reporting

Please select the one below that is the best fit for your research. If you are not sure, read the appropriate sections before making your selection.

☒ Life sciences ☐ Behavioural & social sciences ☐ Ecological, evolutionary & environmental sciences

For a reference copy of the document with all sections, see nature.com/documents/nr-reporting-summary-flat.pdf

Life sciences study design

All studies must disclose on these points even when the disclosure is negative.

Sample size	6 animals per group per time point were used to screen miRNA and mRNA regulation and for histology and immunohistochemistry on whole muscle samples.<br>For isolated muscle progenitor cells, 6 to 9 animals were used per condition per time point for miRNA and mRNA analysis.<br>5 human muscles from independant donors were used to assess muscle progenitor properties.<br>For in vitro functional analysis, between 5 and 25 human muscles from independant donors were used to assess miRNA potential on fibro-adipogenic progenitors cell fate.
Data exclusions	No data were excluded.
Replication	For all figures, multiple independent experiments were performed.<br>All attempts at replicating the observations described in this manuscript were successful.
Randomization	Non-genetically modified C57BL/6 mice were randomly allocated to each treatment/procedure group.<br>All mice were co-housed for at least one week before the beggining of the experiment to reduce cage to cage variations.
Blinding	Investigators were not blinded during experiments or analyses of outcomes. Blinding was not needed for analysis because objective readouts were used in all experiments, and all samples were analyzed in the same way regardless of the groups. In any cases, blinding was not possible due to animals being paraplegic in SCI condition or mobile in SHAM condition.

Reporting for specific materials, systems and methods

We require information from authors about some types of materials, experimental systems and methods used in many studies. Here, indicate whether each material, system or method listed is relevant to your study. If you are not sure if a list item applies to your research, read the appropriate section before selecting a response.

Materials & experimental systems

n/a	Involved in the study
☐	☒ Antibodies
☒	☐ Eukaryotic cell lines
☒	☐ Palaeontology and archaeology
☐	☒ Animals and other organisms
☒	☐ Clinical data
☒	☐ Dual use research of concern

Methods

n/a	Involved in the study
☒	☐ ChIP-seq
☐	☒ Flow cytometry
☒	☐ MRI-based neuroimaging

Antibodies

Antibodies used

Immunohistochemistry :
Rabbit anti-laminin 1:200 ab11575 Abcam
Rabbit anti-Osteocalcin 1:100 ab93876 Abcam
Normal polyclonal Rabbit IgG 1:300 ab-105-c Biotechne
Donkey anti-rabbit Alexa Fluor 594 1:500 R37119 ThermoFisher
Goat anti-rabbit Alexa Fluor 488 1:500 A-11008 ThermoFisher
Rabbit polyclonal anti-Collagen III 1:200 ab7778 Abcam

Cell sorting (mouse) :
Rat IgG2a Isotype FITC 2,5 µL 11-4321-80 Invitrogen
Rat IgG2b Isotype PE 0,5 µL 12-4031-82 Invitrogen
Rat IgG2b Isotype eFluor 450 0,5 µL 48-4031-82 Invitrogen
Rat IgG2a Isotype PerCP-Cy5-5 0,5 µL 45-4321-80 Invitrogen
Rat IgG2a Isotype APC-eFluor 780 1,25 µL 47-4321-82 Invitrogen
Rat IgG2a Isotype PE-Cy7 3 µL 25-4321-82 Invitrogen
Rat IgG2B Isotype Alexa Fluor 647 1 µL IC013R Biotechne
Anti CD34-FITC 2,5 µL 11-0341-82 Invitrogen
Anti CD45-PE 0,5 µL 12-0451-82 Invitrogen
Anti CD31-eFluor450 0,5 µL 48-0311-82 Invitrogen
Anti Sca1-PerCP-Cy5.5 0,5 µL 45-5981-82 Invitrogen
Anti F4/80 APC-eFluor780 1,25 µL 47-4801-82 Invitrogen
Anti CD140a PE-Cy7 3 µL 25-1401-82 Invitrogen
Anti α7 integrin-647 12,5 µL FAB3518R Biotechne

Cell sorting (human) :
PDGFRa biotin 4 µL /106 ₵ BAF322 R&D Systems
CD56 PE 20 µL /106 ₵ 555516 BD Pharmingen
IgG biotin 4 µL /106 ₵ BAF108 R&D Systems
IgG 1 PE mouse 20 µL /106 ₵ A07796 Beckman Coulter
Streptavidine APC/Cy7 1:200 2626040 Sony

Western Blot :
MTOR 1:1000 #29785 CST
OSX 1:500 ab209484 Abcam
SOX9 1:1000 ab185966 Abcam
RUNX2 1:1000 ab192256 Abcam
HRP secondary antibody 1:200000 ab205718 Abcam

Validation

All antibodies were validated by manufacturers for the applications and species used in this study. See manufacturers websites for validation statements.
All antibodies were tested against respective control isotype.

Animals and other research organisms

Policy information about studies involving animals; ARRIVE guidelines recommended for reporting animal research, and Sex and Gender in Research

Laboratory animals

Female CD57BL6 mice, 5-8 weeks

Wild animals

This study did not involve wild animals.

Reporting on sex

Female mice were used because they present less risk of post-operative heterophagy and are easier to handle during urination of paraplegic mice.

Field-collected samples	This study did not involve field-collected samples
Ethics oversight	Mice experimental procedures were approved by the “C2EA-26” Ethics committee in accordance with French Ministry of Research regulations (#25901-2020060515467494).

Note that full information on the approval of the study protocol must also be provided in the manuscript.

Flow Cytometry

Plots

Confirm that:

- [x] The axis labels state the marker and fluorochrome used (e.g. CD4-FITC).
- [x] The axis scales are clearly visible. Include numbers along axes only for bottom left plot of group (a 'group' is an analysis of identical markers).
- [x] All plots are contour plots with outliers or pseudocolor plots.
- [x] A numerical value for number of cells or percentage (with statistics) is provided.

Methodology

Sample preparation	Cellular isolations from mouse muscle were performed following an adapted protocol from Latroche et al.<br>Briefly, gastrocnemius muscles were mechanically minced and incubated in 10 mg/mL collagenase B (11 088 831 001, Roche) with 2,4 U/mL Dispase II (17101015, Gibco) at 37°C for 30 minutes. Digestion was stopped using α-MEM 50% FBS solution and filtered through a 70 µm cell strainer. Red blood cell lysis was performed using commercial buffer (130-094-183, Miltenyi). Cells were incubated in FcBlock (130-092-575, Miltenyi) for 30 minutes and control isotypes and antibodies were added at the indicated concentrations (see antibody section). After incubation, cells were washed and filtered through a 30 µm cell strainer before sorting.<br><br>Muscle samples from patient who underwent chirurgical excision of heterotopic ossifications were finely minced and incubate in Pronase (Merck Cat # 10165921001) solution (1mg/mL final) for 45 minutes at 37°C. The solution was vortexed every 5 minutes before stopping the reaction with 15% FBS medium. Cells were filtered on a 100 µm sieve followed by a 40 µm sieve and put in culture for 2 weeks in a MEM-α, 15% FBS, 1% antibiotics medium.<br><br>Cell sorting was performed on a FACSAria III SORP (BD Biosciences). Cell characterization was performed on a CytoFlex (Beckman Coulter).
Instrument	FACSAria III SORP (BD Biosciences) (cell sorting), CytoFlex (Beckman Coulter) (cell phenotyping)
Software	BD FACSDiva data collection software 7.0, CytExpert data collection Sofware 2.3. Analysis done with FlowJo v10.6.2
Cell population abundance	For mouse cell sorting, abundance are provided on Figure<br>For human cell sorting, abundance of PDGFRα+ CD56- cells varied from 50 to 95% and vice versa for PDGFRα- CD56+ cells whom varied from 5 to 50%. We also noticed the presence of a low abundance PDGFRα+ CD56+ population (<2%).
Gating strategy	Mouse cell sorting :<br>FSC-A/SSC-A -> FSC-A/FSC-H, for single cell gating,<br>Macrophages : CD45+ F4/80+, then on CD45- F4/80- population :<br>Fibro-adipogenic progenitors : CD45- F4/80- Sca1+ CD31- CD34+ CD140+<br>Myogenic progenitors : CD45- F4/80- Sca1- CD31- CD34low α7integrin+<br>Endothelial cells : CD45- F4/80- Sca1+ CD31+ CD34+<br><br>Human cell sorting :<br>Cell sorting (FACSAria III SORP, BD Biosciences) : FSC-A/SSC-A for single cell gating, 7-AAD/SSC-A for cell viability, PDGFRα-Cy7/CD56-PE for cell sorting<br>Cell characterization (CytoFlex, Beckman Coulter) : FSC-A/SSC-A for single cell gating, 7-AAD/SSC-A for cell viability, PDGFRα-Cy7/CD56-PE, CD31-PE/CD34-APC, CD73-PE/CD45-APC, CD105-PE/CD90-APC

- [x] Tick this box to confirm that a figure exemplifying the gating strategy is provided in the Supplementary Information.
